# IL-4 mediated TAP2 downregulation is a dominant and reversible mechanism of immune evasion and immunotherapy resistance in non-small cell lung cancer

**DOI:** 10.1186/s12943-025-02276-z

**Published:** 2025-03-17

**Authors:** Kishu Ranjan, Barani Kumar Rajendran, Imad Ud Deen, Adrien Costantini, Miguel Lopez de Rodas, Shruti S. Desai, Frankie Scallo, Nicole Gianino, Soldano Ferrone, Kurt A. Schalper

**Affiliations:** 1 Department of Pathology, School of Medicine, Brady Memorial Laboratory, Yale University, Room BML 113, New Haven, CT 06520 USA; 2 Department of Surgery, Massachusetts General Hospital and Harvard Medical School, Boston, MA 02114 USA

**Keywords:** HLA class I, TAP2, Biomarkers, CD8 T-cell, CD11b, IL-4Rα, Immunotherapy, Epigenetics

## Abstract

**Background:**

Resistance to both naturally occurring anti-cancer immunity and to immunotherapy is common in patients with aggressive non-small cell lung cancer (NSCLC). Recent studies indicate a role of loss of the HLA class-I antigen presentation machinery (APM) protein β-2-microglobulin in acquired resistance to immune checkpoint blockers. However, the mechanisms, functional consequences and therapeutic potential of APM defects in NSCLC remain poorly understood.

**Methods:**

Using multiplexed immunofluorescence, we spatially mapped CD8^+^ effector Tumor-Infiltrating Lymphocytes (TILs) and the APM components TAP1 and TAP2 in 819 baseline/pre-treatment NSCLCs from patients treated with and without PD-1 axis blockers in 4 independent cohorts. The impact of TAP1/2 silencing in lung cancer cells using siRNAs and CRISPR/Cas9 was studied using transcriptomic analysis, phosphoprotein arrays, ATAC-sequencing, measurement of surface HLA-peptide complexes and in vitro tumor-antigen specific T-cell killing. We established autologous co-cultures of tumor and immune cells from primary human NSCLCs to study the functional impact of IL4Rα and/or PD-1 blockade using monoclonal antibodies. A high-throughput drug screen supported the identification of compounds able to increase TAP2 expression in NSCLC cells.

**Results:**

We identified cancer cell selective TAP2 protein downregulation in 42.4% of treatment naïve NSCLCs associated with reduced sensitivity to immune checkpoint blockers. TAP1 downregulation occurred in 24.4% of lung tumors without survival impact. Silencing of *TAP2* in lung cancer cells altered key intracellular immunomodulatory pathways, limited sensitivity to proinflammatory cytokines, reduced the levels of surface peptide-HLA complexes and protected malignant cells from tumor antigen-specific T-cell killing via SOCS1 upregulation. TAP2 loss in human NSCLCs was associated with reduced *TAP2* promoter chromatin accessibility and elevated IL-4 *IL-4 *expression. Treatment with IL-4 reduced TAP2 levels and the chromatin accessibility of the *TAP2* gene promoter in NSCLC cells and reproduced all the functional consequences of TAP2 loss. In intact human NSCLC, IL-4 *IL-4* transcripts were detected in intratumoral myeloid cells and IL-4Rα blockade increased human NSCLC cell killing by autologous TILs. Epigenetic modulators and other drugs with known anti-cancer activity increased TAP2 expression and its function in lung cancer cells.

**Conclusions:**

Our study reveals previously unrecognized functions of TAP2 beyond antigen presentation and establishes a reversible multi-cellular axis mediating adaptive immune evasion and immunotherapy resistance with clinical potential.

**Supplementary Information:**

The online version contains supplementary material available at 10.1186/s12943-025-02276-z.

## Background

Therapies targeting the inhibitory checkpoints CTLA-4 and PD-1 axis induce prominent anti-tumor responses and lasting clinical benefit in patients with different tumor types, including non-small cell lung cancer (NSCLC). However, most patients do not respond to these treatments and the vast majority of those who initially respond develop acquired resistance [1, 2]. The recognition of cancer cells by T-cells and the therapeutic efficacy of immune checkpoint blockers depend on a fully functional HLA class-I antigen processing and presentation machinery (APM) [3, 4]. Consistent with this notion, deleterious genomic alterations in *β2M* and HLA class-I genes occur in a subset of patients with acquired resistance to immune checkpoint blockers [5–8]. Moreover, the silencing of *β2M* in malignant lung cancer cells is sufficient to confer resistance to PD-1 axis blockers in a syngeneic carcinogen-induced mouse model [6]. Previously, we demonstrated a high frequency of cancer cell selective downregulation of β2M, HLA class-I and HLA class-II proteins in immunotherapy naïve human NSCLCs associated with distinct tumor microenvironment composition and outcomes [9]. In the latter study, the reduced expression of HLA class-I APM proteins in malignant cells was not explained by deleterious mutations in the genes encoding these proteins, suggesting alternative, non-genomic mechanisms mediating these responses [4, 9].

Adequate processing and presentation of immunogenic peptides involve additional APM components beyond HLA class-I proteins, such as immuno-proteasome subunits (e.g. PSMB8, PSMB9, PSMB10), transporters associated with antigen processing (e.g. TAP1 and TAP2) which transport peptides to the endoplasmic reticulum (ER), and key ER chaperones which assist the peptide loading onto the HLA class-I molecules (e.g. Tapasin, Calreticulin, Calnexin and ERp57) [4, 9]. The possible role of these APM components in immune evasion and immunotherapy resistance and the mechanism underlying APM loss in cancer remain poorly understood and have not been exploited diagnostically and/or therapeutically in cancer patients.

Using detailed spatial molecular analysis of human NSCLC cohorts and functional in vitro studies, we demonstrate the dominant effect of cancer cell TAP2 downregulation in tumor immune evasion and immunotherapy resistance. We identify a previously unrecognized multi-step mechanism by which cancer cell TAP2 downregulation mediates adaptive immune escape. We also recognize IL-4 produced in the tumor microenvironment as the causative signal mediating the epigenetic TAP2 silencing in lung cancer. Finally, we perform pharmacologic screens to uncover strategies to revert TAP2 downregulation in lung cancer cells and re-sensitize tumors to T-cell recognition and elimination.

## Material and methods

### Patients, cohorts, and tissue microarrays

Formalin-fixed paraffin-embedded (FFPE) samples from four previously reported retrospective collections of primary NSCLCs represented in tissue microarrays (TMAs) were analyzed [9]. The first collection includes samples from 273 patients with NSCLC collected at Sotiria General Hospital and Patras University General Hospital (Greece) between 1991 and 2001 (cohort #1). Another TMA-based cohort included pre-treatment biopsy or resection samples from 255 patients with NSCLC treated at Yale between 1999 and 2007 (cohort #2). An additional NSCLC collection includes samples from 152 patients with NSCLC seen at Yale Pathology between 1988 and 2012 (cohort #3). NSCLC samples from patients treated with PD-1 axis blockers were retrospectively collected at Yale University (cohort #4, n = 139). All cases in the cohorts were reviewed by a local pathologist using hematoxylin and eosin–stained preparations and tumor histology variant was confirmed by morphology analysis. Tumor cores for TMA construction were obtained from case areas selected by a pathologist to represent the disease and each tumor was represented in 2–4 cores from different sample areas. Tumor core selection was not based on specific tumor segments or location. Clinicopathologic information from patients in all cohorts were collected from clinical records and pathology reports.

### Analysis of public datasets

To determine the frequency and type of mutations in *TAP2* occurring in NSCLC, we performed analysis of publicly available data from The Cancer Genome Atlas (TCGA) lung cancer cohorts. The analysis of variants was performed using paired germline DNA from each case as a reference using the cBioPortal bioinformatic pipeline [10, 11]. Genomic alterations considered to be deleterious included missense mutations, truncating mutations, and deep (e.g., homozygous) deletions. Analysis of the cohorts included both primary lung adenocarcinomas and squamous cell carcinomas. Comparative analysis of mRNA expression of the *TAP1*, *TAP2*, *IFNγ, IL-4,* and *IL-8* from TCGA NSCLC cohorts were conducted using batch-normalized RNA sequencing and transcript levels expressed as RNA-Seq by expectation–maximization units.

### Multiplexed quantitative immunofluorescence (QIF)

Multiplexed immunofluorescence staining protocols for FFPE tissue specimens were developed for simultaneous detection of the cell permeant nuclear dye 4′,6-diamidino-2-phenylindole (DAPI), pancytokeratin (Thermo Fisher, #53–9003-82, clone AE1/AE3), TAP1 (clone NOB1 [12]), TAP2 (clone NOB2 [12]), CD8 (CST, #85336S, clone D8A8Y) and/or HLA-ABC (clone HC-10) using isotype-specific antibodies and different fluorescence conjugates as described previously by our group [9, 13, 14]. For the multiplexed staining, sections were deparaffinized and subjected to antigen retrieval using EDTA Buffer (Sigma-Aldrich; Cat #03690, pH = 8) and boiled for 20 min at 97^0^C in a pressure-boiling container (PT Module, Lab Vision). Slides were then sequentially incubated at room temperature with dual Endogenous Peroxidase Block (Dako, No. S2003) for 10 min and with a blocking solution containing 0.3% BSA in 0.05% Tween solution for 30 min. Secondary antibodies and fluorescence reagents used were anti-mouse Envision (Agilent Technologies, Inc, Cat# K4001), anti-rabbit Envision (Agilent Technologies, Inc, Cat# K4003), rat anti-Mouse IgG1 (eBioscience, #18–4015-82), TSA Cyanine 5 (Akoya Biosciences, Cat# SAT705A001EA), TSA Plus Cyanine 3 (Akoya Biosciences, Cat# NEL744001KT), biotinylated Tyramide/Streptavidin-Alexa750 conjugate (Thermo Fisher Scientific, Cat# S21384). Residual horseradish peroxidase activity between incubations with secondary antibodies was eliminated by exposing the slides twice for 7 min at room temperature to a solution containing benzoic hydrazide (0.136 mg) and hydrogen peroxide (50 μL). To determine the reproducibility of the QIF assay, we measured serial sections from the index TMA containing positive and negative controls at different timepoints.

### Tissue fluorescence measurement and scoring

Quantitative measurement of the fluorescence signal was performed using the AQUA® method that enables objective and sensitive measurement of targets within user-defined tissue compartments [9, 13, 15]. The TMA slides were scanned using an automated HistoRx PM2000 multispectral slide scanner with 20 × magnification and autoexposure configuration. Briefly, the QIF score of each target in the cytokeratin positive (CK^+^) cancer cell compartment was calculated by dividing the target pixel intensities by the area of CK positivity. Scores were normalized to the exposure time at which the images were captured, allowing scores collected at different exposure times to be comparable. Markers were also measured in the CK-negative stromal cell tissue compartment by collecting the signal score in the area defined by DAPI staining and lacking CK^+^ pixels. Cases were considered to have TAP2 protein downregulation (e.g. TAP2 low) when the signal in CK^+^ cancer cells was lower than in the neighboring CK-negative (non-tumor) stromal cells within the same sample. The specific marker staining patterns and cancer cell–specific loss was confirmed by visual inspection by trained personnel.

### In situ mRNA measurement using QIF

Multiplexed QIF protocols for FFPE tissue specimens were developed for simultaneous detection of the *IL-4* mRNA, *CD11b* mRNA, TAP2 protein and cytokeratin (CK) protein using the RNAscope™ assay coupled to multiplex immunofluorescence. In brief, the FFPE tissue sections were deparaffinized, endogenous peroxidase activity was quenched with hydrogen peroxide followed by target retrieval and protease plus treatment. The in situ mRNA detection was performed using the RNAscope™ Multiplex Fluorescent V2 kit containing *IL-4* (Advanced Cell Diagnostics, Inc., Cat# 315,191) and *CD11b* (Advanced Cell Diagnostics, Inc., Cat# 555,091) mRNA detection probes in C1 and C2, respectively. Post RNA probing, the slides were sequentially stained for TAP2, and CK proteins followed by DAPI nuclear staining. The TMA slides were scanned using an automated Akoya Vectra Polaris slide scanner with 20 × magnification and autoexposure configuration. Quantitative measurement of the fluorescence signal was performed using the AQUA® method [9].

### Cell culture and transfection

The lung adenocarcinoma cell lines A549 (HLA-A2^+^, KRAS mutant), H1975 (HLA-A2^−^, EGFR mutant), and H520 (HLA-A2^−^, KRAS/EGFR wild type) were purchased from the American Type Culture Collection (ATCC). PC9 (HLA-A2+, EGFR mutant) cell line was purchased from Sigma-Aldrich (Cat# 90071810-1VL). PC9, H1975, and H520 cells were grown in RPMI-1640 culture media and A549 cells were grown in DMEM culture media. All the culture media were supplemented with 10% fetal bovine serum and Penicillin–streptomycin antibiotic cocktails. Cells were incubated in a humidified incubator supplied with 5% CO_2_. Exponentially grown cells were used in this study. Cell lines were authenticated every 3–6 months using the GenePrint® 10 System in the Yale University DNA Analysis Facility. Cells were also periodically tested for mycoplasma contamination.

To mimic cancer cell downregulation of TAP1 & TAP2, we transiently reduced TAP1 and TAP2 expression in A549 and PC9 cells. Briefly, cells were transfected with 100 nM scrambled ON-TARGETplus SMARTpool small interference RNA (siRNA) against TAP1 (Cat# L-007634–00-0005) and/or TAP2 (Cat# L-007635–00-0005) (4 pooled siRNAs for each gene, Horizon Discovery Biosciences Limited, USA) using lipofectamine RNAiMAX reagent (Invitrogen, Cat# 13778150). Forty eight h after transfection, the knockdown efficacy was confirmed at RNA and protein levels. Data shown in supplementary Figs. S3A-E.

For overexpression studies, 250 ng of N-terminal FLAG tag TAP2 constructs (Genecopoeia, Cat# EX-Z2364-M11) were transfected to A549 cells using lipofectamine 3000 reagent (Invitrogen, Cat# L3000008). Cells were incubated for 48 h. Transfection efficiency was confirmed by intracellular staining using anti FLAG antibody (CST, Cat# 8146S) and subsequent analysis was performed using FCS Express software version 7 (De Novo software). Data shown in supplementary Fig. S3F.

For TAP2 re-expression in TAP2 knockout cells, 250 ng of N-terminal FLAG tag TAP2 constructs were transfected to A549 TAP2 knockout cells using lipofectamine 3000 reagent followed by incubation for 48 h. Expression of FLAG-TAP2 was confirmed by intracellular staining using anti FLAG antibody (CST, Cat# 8146S) and subsequent analysis was performed using FCS Express software version 7 (De Novo software).

### Generation of TAP2 knockout (TAP2 KO) cells

A549 cells were transfected with pSpCas9 BB-2A-GFP (GenScript, Cat# SC1678) containing *TAP2* targeting gRNA sequence 5’-GCGCCTTGTACCTGCTCGTA-3’. Cells were incubated for 48 h followed by screening of GFP positive cells using cell sorter (BD Biosciences). GFP positive cells were serially diluted to obtain monoclonal cell populations. Monoclonal *TAP2* knockout cell clones were confirmed by TAP2 protein expression and DNA sequencing. Data shown in supplementary Fig. 2I.

### Quantitative Real time PCR (qRT-PCR)

The total mRNA was isolated using Trizol reagent (Thermo Fisher Scientific Cat# 15,596,026). cDNA was prepared from 1 µg of total mRNA using iScript cDNA Synthesis Kit (Bio-Rad, Cat#1,708,891) and qRT-PCR was performed using iQ SYBR Green Super Mix (Bio-Rad, Cat #1,708,880) with normalization to *GAPDH*. Primer sequences are described in the supplementary Table S1. Data shown in supplementary Fig. S3A & 3C.

### Immunoblotting

Cells were washed with ice cold PBS and lysed in 1X RIPA buffer (Thermo Fisher, Cat# 89,900) supplemented with protease inhibitors (Thermo Fisher, Cat# 78,440). Immunoblotting was performed with antibodies to detect TAP1 (1:100, Cat# NOB1), TAP2 (abcam,1:500, Cat# ab180611), GAPDH (1:10,000, EMD Millipore, clone 6C5, Cat# CB1001) and fluorophore conjugated secondary antibodies DyLight 680 Goat Anti-Mouse IgG (H + L) (1:10,000, Thermo Fisher. Cat# 35,519), DyLight 680 Goat Anti-Rabbit IgG (H + L) (1:10,000, Thermo Fisher. Cat# 35,569) and DyLight 800 Goat Anti-Mouse IgG (H + L) (1:10,000, Thermo Fisher. Cat# SA510176). Blots were scanned using Licor odyssey (LI-COR Biotechnology, USA). Data shown in supplementary Fig. S3E.

#### NanoString analysis

The differential expression analysis of 770 immune related genes was performed using the nCounter PanCancer Immune Profiling panel (NanoString Technologies). A549 cells were transfected with 100 nM scrambled ON-TARGETplus SMARTpool small interference RNA (siRNA) against TAP2 (Cat# L-007635–00-0005) (4 pooled siRNAs for each gene, Horizon Discovery Biosciences Limited, USA) using lipofectamine™ RNAiMAX transfection reagent (Invitrogen). After 48 h of transfection cells were treated with IFNγ alone or IFNγ + TNFα for 8 h. After treatments, the total mRNA was isolated using Trizol reagent (Thermo Fisher Scientific Cat# 15,596,026) and the purity of each RNA sample was determined using a NanoDrop spectrophotometer (Thermo Fisher Scientific). A total of 200 ng mRNA was hybridized with PanCancer reporter probes (NanoString, Cat# 100,054) included in the 770-plex PanCancer Immune Profiling panel (NanoString, Cat# 115,000,132). Post hybridization, samples were placed into the nCounter MAX/FLEX System Prep Station (NanoString Technologies) to measure RNA counts. Finally, the RNA counts were normalized for technical efficiency by the geometric mean of internal control probes using nSolver 4.0 Advanced Analysis software (NanoString Technologies). The differential expression data shown represents normalized counts transformed to log_2_ scale.

### Human Phospho-Kinase Array

A549 cells were transfected with 100 nM scrambled ON-TARGETplus SMARTpool small interference RNA (siRNA) against TAP2 (Cat# L-007635–00-0005) (4 pooled siRNAs for each gene, Horizon Discovery Biosciences Limited, USA) using lipofectamine™ RNAiMAX transfection reagent (Invitrogen). After 48 h of transfection the cells were treated with IFNγ + TNFα (20 ng/ml each) for 24 h. Post incubation, the expression levels of 37 phospho kinases were examined using the Proteome Profiler Human Phospho-Kinase Array Kit (R&D Systems, Inc., Cat# ARY003C) according to the manufacturer’s instructions. Data shown includes the top 5 upregulated and top 5 downregulated phosphoproteins.

### HLA-peptide stripping and peptide reloading assay

To unload peptides bound to surface HLA, cells (2 × 10^6^/ml) were incubated with citric acid-Na_2_HPO_4_ buffer (0.263 M citric acid and 0.123 M Na2HPO4 containing 1% BSA (pH 3)) for 2 min and washed three times with 5 ml of culture medium before analysis by flow cytometry. To reload peptides into the surface HLA, cells were incubated with purified HER2_369-377_ (AnaSpec Inc., Cat# SQ-ASPE 77814) or MAGE3_271-279_ peptides (AnaSpec Inc., Cat# AS-61355) (50 µM and 100 µM) in a CO_2_ incubator for 2 h. Finally, cells were washed and stained using mAb 4F10 specific to HLA-A2 bound to the HER2 nonamer peptide KIFGSLAFL (corresponding to amino acids 369–377 of the full-length HER2 protein (HER2_369-377_)) [16] or mAb 12B6 specific to HLA-A2 bound to the MAGE3 nonamer peptide FLWGPRALV (corresponding to amino acids 271–279 of the full-length MAGE3 protein (MAGE3_271-279_)) [17] and analyzed with a LSR II flow cytometer (BD Biosciences). Subsequent analysis was performed using FCS Express software version 7 (De Novo software). Data shown in supplementary Figs. S2A-E.

### Flow cytometry analysis

For surface staining, A549, PC9, H1975 and H520 cells were washed two times with FACS buffer (2% fetal bovine serum + 0.5% bovine serum albumin) followed by incubation with antibodies targeting HLA-A2-HER2_369-377_ (SB21-34, 4F10) [16] or HLA-A2-MAGE3_271-279_ (SB21-33, 12B6) [17] for 30 min in ice. Cells were washed with FACS buffer and further incubated with anti-mouse alexa fluor® 647 conjugate (CST, Cat# 4410) secondary antibody and evaluated on a LSR II flow cytometer (BD Biosciences).

For intracellular staining, control and treated cells were fixed with 4%PFA for 15 min and permeabilized with 100% methanol for 30 min on ice. Cells were washed three times with FACS buffer followed by incubation with antibodies targeting TAP1 (NOB1), TAP2 (abcam, Cat# ab180611) and SOCS1 (abcam, Cat# ab9870) for 1 h in ice. Cells were washed three times with FACS buffer followed by staining with anti-rabbit alexa fluor® 647 Conjugate (CST, Cat #4414) or donkey anti-Goat alexa fluor® 488 (abcam, ab150129) secondary antibodies and evaluated on a LSR II flow cytometer (BD Biosciences). Subsequent analysis was performed using FCS Express software version 7 (De Novo software).

### Primary NSCLC samples and single cell isolation

Fresh human lung tumor samples were washed with RPMI-1640 culture medium, cut into small pieces of approximately 2–3 mm^3^ and incubated with tissue digestion enzyme mix (Miltenyi Biotec, Cat#130–108-339) for processing in a gentleMACS™ Dissociator (Miltenyi Biotec.) for 20 min. The digested tissue suspension was passed through a cell strainer and centrifuged at 300 g for 7 min. The acquired cell pellet was dissolved in RPMI-1640 medium and cell viability was measured using 0.4% trypan blue solution. In selected experiments, CD3 T-cells were eliminated from the tumor single cell suspension using CD3 depleting microbeads (Miltenyi Biotec, Cat# 130–050-101) as per manufacturer instructions.

For flow cytometry analysis, the tumor single cell suspensions were incubated with Fc blocker (Biolegend, Cat# 422,302) for 15 min and then incubated with EpCAM (Biolegend, Cat# 324,252), CD3 (BD Biosciences, Cat# 555,333), Annexin-V (Biolegend, Cat# 640,922), CD8 (BD Biosciences, Cat# 566,852) CD11b (BD Biosciences, Cat# 563,839) and CD25 (BD Biosciences, Cat# 560,987) antibodies for 30 min in ice. Cells were acquired using an LSR II flow cytometer (BD Biosciences). Subsequent analysis was performed using FCS Express software version 7 (De Novo software).

### ATAC-seq and RNA-seq

Single cell suspensions from primary human lung tumors or cell lines were analyzed using assay for transposase-accessible chromatin using sequencing (ATAC-seq) and whole transcriptome sequencing (RNA-seq) using standard protocols at the Yale Center for Genome Analysis (YCGA). Bulk ATAC-seq reads were trimmed using TrimGalore (https://github.com/FelixKrueger/TrimGalore) and aligned to the human genome (Hg38 assembly) using Bowtie2 (v2.3.4.3) [18]. Sambamba (v0.7.1) [19] was used to sort the BAM file and unpaired reads were removed. PCR duplicates were removed using Picard (v1.31) mark duplicates function (http://broadinstitute.github.io/picard/) and mitochondrial reads were removed using Samtools (v.1.12) view function [20]. All downstream analyses were performed on these filtered reads. Chromatin accessibility peak enrichment analysis and differential mean peak levels across specimens were identified using MACS2 (V2.2.7.1) [21] with default parameters except -nomodel, extsize 200 -SPMR. Further filtering of enriched peaks was performed using adjusted p-value < 0.05 and fold enrichment score. Differentially enriched peaks were annotated using ChIPseeker R package (https://bioconductor.org/packages/release/bioc/html/ChIPseeker.html). Gene regions flanking the transcription start site (+ 3 kb) and transcription end site (-3 kb) of selected genes were considered for the analysis of promoter regions and enriched differential peaks were used to identify predicted transcription factor binding sites using the motif discovery function in MEME Suite (https://meme-suite.org/meme/). Motifs identified in the *TAP2* promoter were used for further identification of transcription factors and their predicted binding sites using AnimalTFDB (v3.0) (https://guolab.wchscu.cn/hTFtarget/#!/). Predicted transcription factors were further filtered based on q-value < 0.05 and high binding score. For the purpose of visualization, genome coverage files from filtered BAM files were generated using BEDtools (v2.30.0) [22] and each position was normalized by dividing the total library size and multiplying by 106. Further, BEDgraph (Bdg) files were converted into BigWig format using the bedGraphToBigWig command from the UCSC genome browser tool. Integrated genome viewer (IGV) (v2.13.2) was used for peaks visualization and comparison. The mRNA transcripts in paired preparations from non-tumor lung tissue and NSCLC were analyzed using TrimGalore (v.0.6.7). Trimmed FASTQ files were evaluated using MultiQC (v.1.10.1). The sequence reads were mapped to the Human genome version Hg38 using HISAT2(v.2.2.1) and SubRead (v.2.0.3) tool was used to quantify the gene expression using featureCount function [23]. Gene read counts were normalized using edgeR package (v.3.38.4) [24]. DESeq2 R package (v.1.36.0) was employed for differential expression analysis. Differentially expressed genes (DEGs) were selected from the normalized counts (using log2 counts per million reads (CPM) between non-tumor lung tissue and NSCLC samples (with significant fold change > 1.5 and false discovery rate < 0.05) [25].

### Ex vivo tumor cell killing assay using autologous tumor/immune cell co-cultures

Single cell suspensions from primary NSCLCs were incubated for 16 h in a CO_2_ incubator followed by stimulation with IFNγ + TNFα (20 ng/ml each) or recombinant IL-4 (20 ng/ml) for 24 h. Post incubation, cells were treated with anti-IL-4Rα antibody (R&D Systems, Cat# MAB230-100) (5 µg/ml) and/or pembrolizumab/PD-1 blocking antibody (Selleck Chemicals, Cat# A2005) (5 µg/ml) for an additional 72 h. To analyze cancer cell death, the preparations were stained with lineage specific or functional surface markers (EpCAM, Annexin-V, CD8, CD25, as mentioned above) and acquired using an LSR II flow cytometer (BD Biosciences). Subsequent analysis was performed using FCS Express software version 7 (De Novo software).

### In vitro tumor antigen specific killing, cytotoxicity and cell viability assays

Target tumor cells were either transfected with scrambled siRNA or siRNA targeting *TAP1* (siTAP1), *TAP2* (siTAP2) and *TAP1* with *TAP2* (siTAP1 + TAP2) using lipofectamine™ RNAiMAX transfection reagent (Invitrogen). Forty eight h after transfection, cells were stimulated with IFNγ or IFNγ + TNFα for an additional 24 h, followed by incubation with effector anti-HER2_369-377_ CD8 T-cells (Cellero, Cat# ASTC-1126) at 0:1, 2:1 and 5:1 effector-to-target cell ratio for 72 h. Annexin V staining was carried out using a FITC Annexin V Apoptosis Detection Kit (Biolegend, Cat# 640,922) and analyzed by flow cytometry. Cytotoxicity assay was carried out using CyQUANT™ LDH Cytotoxicity Assay Kit (Thermo Fisher Scientific, Cat#C20302) as per manufacturer’s instruction. The LDH fluorescence signal in cell plates was acquired using a Tecan microplate reader and the data was represented as percentage of LDH release. Cell viability assay was analyzed using 96 well plates with the CyQUANT™ MTT Cell Viability Assay (Thermo Fisher Scientific, Cat# V13154). Cell plates were acquired and analyzed using a Tecan microplate reader and data was represented as percentage of viable cells. A549 or PC9 cells were treated with SAHA/Vorinostat (Selleckchem.com, Cat# S1047), BML-281 (Avantor®, Cat#76002-128) or WT161 (Cayman Chemical, Cat# 21099), and analyzed for Annexin V staining, cytotoxicity/LDH release and cell viability/MTT.  

### High throughput drug screening

Changes in TAP2 protein levels in A549 cells were screened with the Pharmakon-1600, Enzo 640 FDA-approved drugs (Microsource Discovery Systems) and Enzo Epigenetics library available at the Yale Center for Molecular Discovery (YCMD). Briefly, 2,000 cells/well were plated in 20 µl volume of glass bottom 384-well imaging plates (Cellvis, P384-1.5H-N) and incubated at 37ºC for 24 h. Twenty nL of 10 mM DMSO stock of each screening compound (or DMSO used as vehicle control) was transferred using an Echo Acoustic Dispenser (Labcyte) to assay plates containing 20 µl A549 cells for a final screening compound concentration of 10 µM (0.1% DMSO final). After 24 h of compound treatment, cells were washed with 30 µl of PBS and fixed with 4% (v/v) paraformaldehyde (Electron Microscopy Sciences 15,710-S) diluted in PBS at room temperature for 20 min. Cells were washed twice with 30 µl PBS, then permeabilized with 30 µl of 0.5% (v/v) Triton X-100 in PBS for 5 min. Following fixation and permeabilization, cells were washed twice with 30 µl PBS and incubated with 30 µl of blocking buffer consisting of 10% (v/v) FBS (Gibco, 16,140–071) diluted in PBS, for 1 h at room temperature. Primary antibody solution was prepared by diluting TAP2 (TAP2, Abcam Cat# ab180611) and PD-L1 antibodies (CST, clone E1L3N, Cat# 13684S) to 1:250 (v/v) in blocking buffer. After blocking, cells were incubated with 20 µl of antibody solution (either TAP2 or PD-L1) at 4ºC overnight. Next day, cells were washed twice with 30 µl PBS and incubated with 20 µl secondary antibody solution, consisting of 1:1000 (v/v) goat anti-rabbit alexa fluor 488 (Invitrogen A11034), 1:1000 Hoechst 33,342 dye (Invitrogen, H1399) and 1:5000 HCS CellMask Deep Red Stain (Invitrogen, H32721) in blocking buffer, for 1 h in the dark at room temperature. Cells were washed twice with 30 µl PBS and 50 µl of PBS was added to each well and scanned with a GE Healthcare IN Cell Analyzer 2200. Image analysis was conducted using a custom pipeline in CellProfiler 3.1.9 (www.cellprofiler.org). Activity cut-off for hit selection was determined by using three standard deviations from the mean of the whole sample population.

### Statistical analysis

**Fig. 1:**
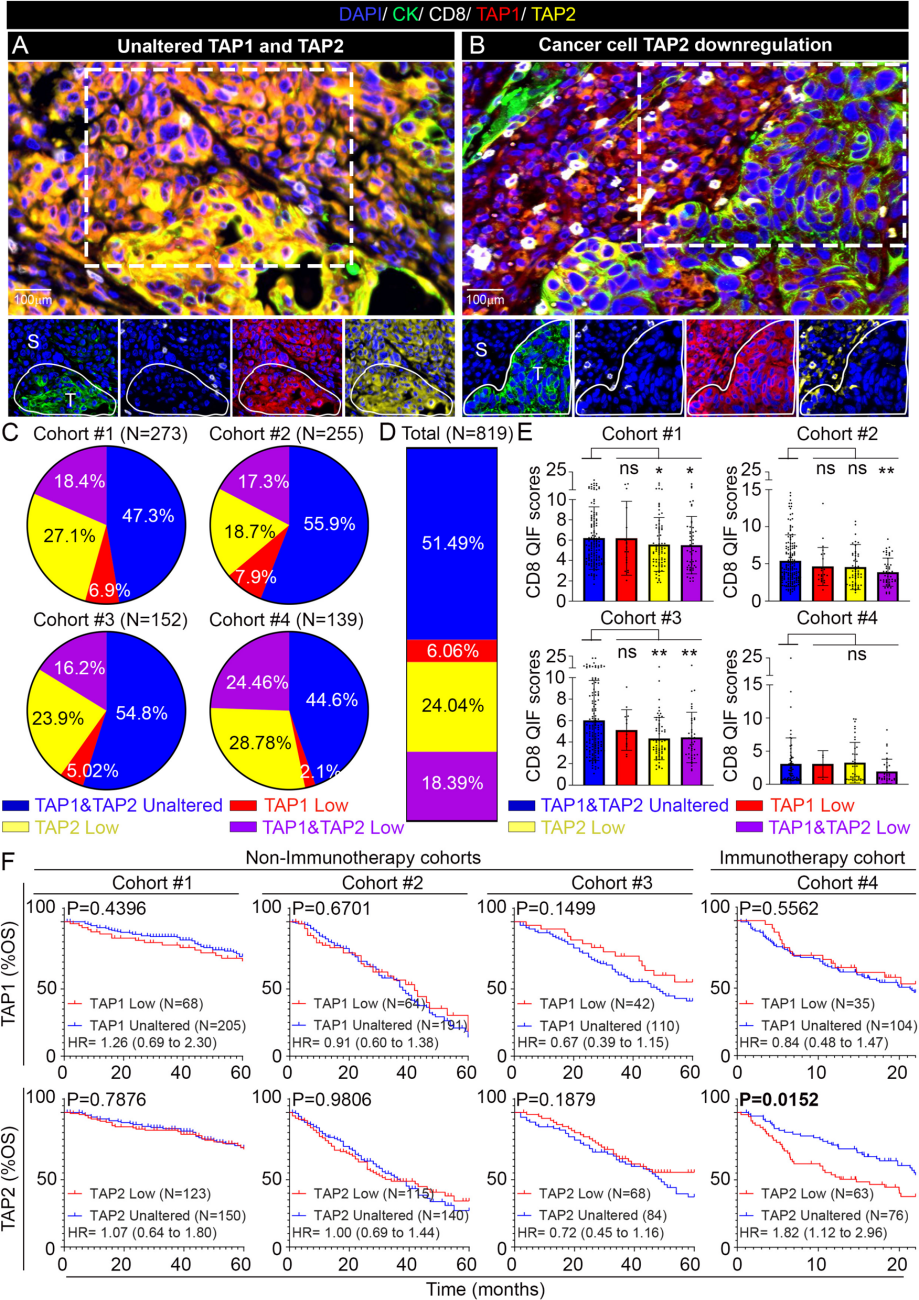
Cancer cell selective downregulation of TAP2 is frequent and associated with worse overall survival in NSCLC patients treated with immunotherapy. **A**, **B** Representative multicolor immunofluorescence microphotographs showing the simultaneous detection of CK^+^ tumor epithelial cells (green), CD8^+^ TILs (white), TAP1 (red) and TAP2 (yellow) proteins in human NSCLC. Cell nuclei were stained with DAPI (blue). **C** Pie charts showing the frequency of cancer cell TAP1 and/or TAP2 downregulation in baseline samples from 4 cohorts of NSCLC patients treated with chemotherapy (Cohorts #1, #2 and #3) or using PD-1 axis blockers (Cohort #4). **D** Mean frequency of cancer cell TAP1 and/or TAP2 downregulation across 4 NSCLC cohorts. **E** Mean levels of CD8^+^ TILs in cases from NSCLC cohorts #1–4. CD8 QIF scores are shown as thousands of fluorescence units. **F** Kaplan–Meier graphical analysis of the overall survival (OS) of patients from cohorts #1–4 stratified by the presence or absence of cancer cell TAP1 (upper panels) and TAP2 (lower panels) downregulation. Data presented as the mean ± s.d. For E, *, *p* < 0.05; **, *p* < 0.01 determined by two-tailed unpaired Student’s t-test. F, log-rank test. ns, not significant; HR, hazard ratio; OS, overall survival; QIF, quantitative immunofluorescence; S, stroma; T, tumor; TILs, tumor infiltrating lymphocytes

QIF signals between markers and compartments were analyzed using the mean fluorescence scores and compared using non-parametric Mann–Whitney test. Patient characteristics were compared using the Student’s t-test for continuous variables and χ2 test for categorical variables. Survival functions were compared using Kaplan–Meier estimates and statistical significance was determined using the log-rank test. Stratification of cases for survival analysis was conducted based on the frequency of cancer cell TAP1/2 downregulation across the cohorts (25^th^ percentile for TAP1 and the 45^th^ percentile for TAP2, Fig. 1D). The same cut-points were used in all the cohorts. For comparisons across experiments using flow cytometry analysis and other molecular measurements, the Student’s t-test was used. All the statistical analyses were conducted using JMP Pro. v11 and GraphPad Prism v7.0a software. Statistical significance between groups was determined using a two-tailed p-value < 0.05.

## Results

### Cancer cell selective downregulation of TAP2 is frequent in NSCLC

Using multiplex quantitative immunofluorescence (QIF), we studied the levels and spatial distribution of TAP1 and TAP2 proteins in 819 pre-treatment NSCLCs from 4 independent cohorts of patients treated with chemotherapy and/or surgery (cohort #1, *n* = 273; cohort #2, *n* = 255 and cohort #3, *n* = 152, Tables 1–2) or treated with immune checkpoint blockers (cohort 4, *n* = 139, Table 2) represented in tissue microarrays. As shown in Fig. 1A, most tumors showed comparable levels of TAP1 and TAP2 protein in both CK-positive cancer cells and in CK-negative (non-malignant) stromal cells. However, a subset of cases showed marked reduction of TAP1 and/or TAP2 only in cancer cells (CK^+^) with preservation of the signal in the neighboring non-tumor lung tissue and/or stromal cells (CK^-^) supporting cancer cell selective protein downregulation (Fig. 1B and Supplementary Fig. S1A-B). As shown in Fig. 1C and Fig. 1D, and using automated scoring of the TAP1/2 protein signal in cancer cells and in adjacent non-tumor cells, we found 6.1% of cases showing TAP1 downregulation (range 2.1–7.9% in Cohorts #1–4), 24.0% with TAP2 downregulation (range 18.7–28.8% in Cohorts #1–4) and 18.4% with concurrent TAP1 and TAP2 downregulation (range 16.2–24.5% in Cohorts #1–4).

**Table 1:** Association of TAP1/2 expression (non-immunotherapy treated cohort #1 and cohort #2) with clinicopathologic variables. Statistical analysis by Chi-Square test

	**Cohort #1**									**Cohort #2**								
	**TAP1/2 unaltered**	**TAP1 low**	**p value**	**TAP1/2 unaltered**	**TAP2 low**	**p value**	**TAP1/2 unaltered**	**TAP1/2 low**	**p value**	**TAP1/2 unaltered**	**TAP1 low**	**p value**	**TAP1/2 unaltered**	**TAP2 low**	**p value**	**TAP1/2 unaltered**	**TAP1/2 low**	**p value**
**Age**																		
≤ 70	66 (87%)	10 (13%)	0.826	66 (43%)	38 (25%)	**0.026**	66 (69%)	39 (31%)	0.693	114 (86%)	19 (16%)	0.943	114 (77%)	35 (23%)	0.579	114 (76%)	37 (24%)	0.659
> 70	59 (88%)	8 (12%)		59 (50%)	38 (32%)		59 (72%)	13 (28%)		23 (85%)	4 (15%)		23 (72%)	9 (28%)		23 (79%)	6 (21%)	
**Sex**																		
Male	48 (84%)	9 (16%)	0.381	48 (64%)	27 (36%)	0.840	48 (69%)	22 (31%)	0.693	128 (86%)	21 (14%)	0.566	128 (74%)	44 (26%)	0.103	128 (77%)	38 (23%)	0.290
Female	82 (89%)	10 (11%)		82 (63%)	49 (37%)		82 (72%)	33 (28%)		19 (90%)	2 (10%)		19 (91%)	2 (9%)		19 (68%)	9 (32%)	
**Stage**																		
Stage I-II	116 (86%)	19 (14%)	0.132	116 (61%)	75 (39%)	**0.011**	116 (69%)	52 (31%)	0.252	90 (86%)	15 (14%)	0.714	90 (74%)	32 (26%)	0.305	90 (78%)	26 (22%)	0.472
Stage III-IV	14 (100%)	0 (0%)		14 (93%)	1 (7%)		14 (82%)	3 (18%)		57 (88%)	8 (12%)		57 (80%)	14 (20%)		57 (73%)	21 (27%)	
**Histology**																		
ADC	109 (89%)	13 (11%)	0.093	109 (69%)	49 (31%)	**0.0007**	109 (76%)	34 (24%)	**0.0001**	61 (87%)	9 (13%)	0.516	61 (85%)	11 (15%)	**0.011**	61 (72%)	24 (28%)	0.512
SCC	16 (76%)	5 (24%)		16 (40%)	24 (60%)		16 (44%)	21 (56%)		67 (91%)	7 (9%)		67 (68%)	32 (32%)		67 (76%)	21 (24%)	
**Smoking**																		
No/Former	100 (88%)	14 (12%)	0.311	100 (63%)	60 (37%)	0.336	100 (71%)	40 (29%)	0.608	34 (87%)	5 (13%)	0.882	34 (79%)	9 (21%)	0.612	34 (81%)	8 (19%)	0.376
Smoker	30 (86%)	5 (14%)		30 (66%)	16 (34%)		30 (67%)	15 (33%)		113 (86%)	18 (14%)		113 (75%)	37 (25%)		113 (74%)	39 (26%)	
**Mutation**																		
* EGFR*	Not available									Not available								
* KRAS*																		
* EGFR/KRAS*-WT																		

Bold text indicates *p* < 0.05. *WT* wild type, *ADC* adenocarcinoma, *SCC* squamous cell carcinoma

**Table 2:** Association of TAP1/2 expression (non-immunotherapy treated cohort #3 and immunotherapy treated cohort #4;) with clinicopathologic variables. Statistical analysis by Chi-Square test

	**Cohort #3**									**Cohort #4**								
	**TAP1/2 unaltered**	**TAP1 low**	**p value**	**TAP1/2 unaltered**	**TAP2 low**	**p value**	**TAP1/2 unaltered**	**TAP1/2 low**	**p value**	**TAP1/2 unaltered**	**TAP1 low**	**p value**	**TAP1/2 unaltered**	**TAP2 low**	**p value**	**TAP1/2 unaltered**	**TAP1/2 low**	**p value**
**Age**																		
≤ 70	56 (88%)	8 (12%)	0.266	56 (67%)	27 (33%)	0.802	56 (78%)	16 (22%)	0.499	38 (97%)	1 (3%)	0.571	38 (73%)	14 (27%)	0.391	38 (70%)	16 (30%)	0.635
> 70	34 (94%)	2 (6%)		34 (65%)	18 (35%)		34 (72%)	13 (28%)		38 (95%)	2 (5%)		38 (66%)	20 (34%)		38 (75%)	13 (25%)	
**Sex**																		
Male	39 (89%)	5 (11%)	0.624	39 (64%)	22 (36%)	0.510	39 (75%)	13 (25%)	0.783	33 (97%)	1 (3%)	0.673	33 (59%)	22 (41%)	0.286	33 (67%)	16 (33%)	0.578
Female	54 (92%)	5 (8%)		54 (69%)	24 (31%)		54 (77%)	16 (23%)		29 (94%)	2 (6%)		29 (62%)	18 (38%)		29 (62%)	18 (38%)	
**Stage**																		
Stage I-II	10 (83%)	2 (17%)	0.447	10 (59%)	7 (41%)	0.548	10 (91%)	1 (9%)	0.142	2 (100%)	0 (0%)	0.316	2 (33%)	4 (67%)	0.155	2 (67%)	1 (33%)	0.076
Stage III-IV	3 (100%)	0 (0%)		3 (75%)	1 (25%)		3 (60%)	2 (40%)		60 (95%)	3 (5%)		60 (63%)	36 (37%)		60 (65%)	33 (35%)	
**Histology**																		
ADC	65 (93%)	5 (7%)	0.242	65 (70%)	28 (30%)	0.096	65 (83%)	13 (17%)	**0.005**	45 (94%)	3 (6%)	0.770	45 (65%)	24 (35%)	**0.027**	45 (67%)	22 (33%)	0.179
SCC	16 (84%)	3 (16%)		16 (53%)	14 (47%)		16 (57%)	12 (43%)		9 (100%)	0 (0%)		9 (39%)	14 (61%)		9 (50%)	9 (50%)	
**Smoking**																		
No/Former	67 (89%)	8 (11%)	0.591	67 (65%)	36 (35%)	0.431	67 (78%)	19 (22%)	0.501	45 (98%)	1 (2%)	0.226	45 (56%)	35 (44%)	0.787	45 (59%)	31 (41%)	0.148
Smoker	26 (93%)	2 (7%)		26 (72%)	10 (28%)		26 (72%)	10 (27%)		9 (90%)	1 (10%)		9 (60%)	6 (40%)		9 (82%)	2 (18%)	
**Mutation**																		
* EGFR*	Not available									6 (100%)	0 (2%)	0.543	6 (75%)	2 (25%)	0.360	6 (86%)	1 (14%)	0.228
* KRAS*										16 (94%)	1 (6%)		16 (57%)	12 (43%)		16 (62%)	10 (38%)	
* EGFR/KRAS*-WT										43 (96%)	2 (4%)		43 (70%)	18 (30%)		43 (66%)	22 (34%)	

Bold text indicates *p* < 0.05. *WT* wild type, *ADC* adenocarcinoma, *SCC* squamous cell carcinoma

### TAP2 downregulation is associated with reduced sensitivity to immunotherapy

Tumors with downregulation of TAP1, TAP2 or TAP1 & TAP2 showed mild and inconsistent differences in the levels of CD8^+^ TILs relative to specimens with unaltered TAP1/2 expression. As shown in Fig. 1E, tumors with TAP2 downregulation alone showed a trend towards lower CD8^+^ TILs (range 6.6–33.3%) that reached statistical significance only in cohorts #1 and #3. Similar differences were seen in cases with TAP1 & TAP2 downregulation, and no differences were seen in cases with TAP1 downregulation alone. In NSCLCs from cohorts #1 and #2 the levels of HLA class-I proteins (e.g. HLA-ABC) in malignant cells were comparable across cases with TAP1 and/or TAP2 downregulation (Supplementary Fig. S1C). The levels of cancer cell PD-L1 protein were lower in cases with TAP1/2 downregulation in cohort #1, but no difference was seen in cohorts #2 and #4, suggesting independence of TAP1/2 loss from other adaptive immune evasion mechanisms (Supplementary Fig.S1D).

In the analysis of clinicopathologic variables, cases with low TAP1 showed no consistent associations and TAP2 downregulation was more frequent in tumors with squamous cell histology than in adenocarcinomas (Tables 1–2). No consistent associations between TAP1 or TAP2 downregulation and age, gender, disease stage or smoking status were identified, and the frequency of cancer cell TAP1 and/or TAP2 downregulation was comparable across lung adenocarcinomas harboring activating mutations in EGFR or KRAS (Tables 1–2). These results indicate that TAP1 and TAP2 protein downregulation do not strongly segregate with specific patient/tumor characteristics and can occur in any NSCLC subtype.

To determine the impact of baseline TAP1 and TAP2 downregulation in treatment-specific outcomes, we studied the overall survival (OS) of patients in the NSCLC cohorts treated with standard chemotherapy and/or surgery (cohorts #1, #2 and #3) or after treatment with PD-1 axis blockers (cohort #4). As shown in Fig. 1F, patients with tumors containing low cancer cell TAP1 showed comparable OS with those without TAP1 downregulation regardless of the treatment type. However, low cancer cell TAP2 expression was associated with a significantly shorter OS and 82% increased risk of death only in patients treated with PD-1 axis blockers (Fig. 1F) (Cohort #4; HR 1.82 [CI: 1.12–2.96]).

### TAP2 expression modulates surface HLA class-I antigen complexes in lung cancer cells

**Fig. 2:**
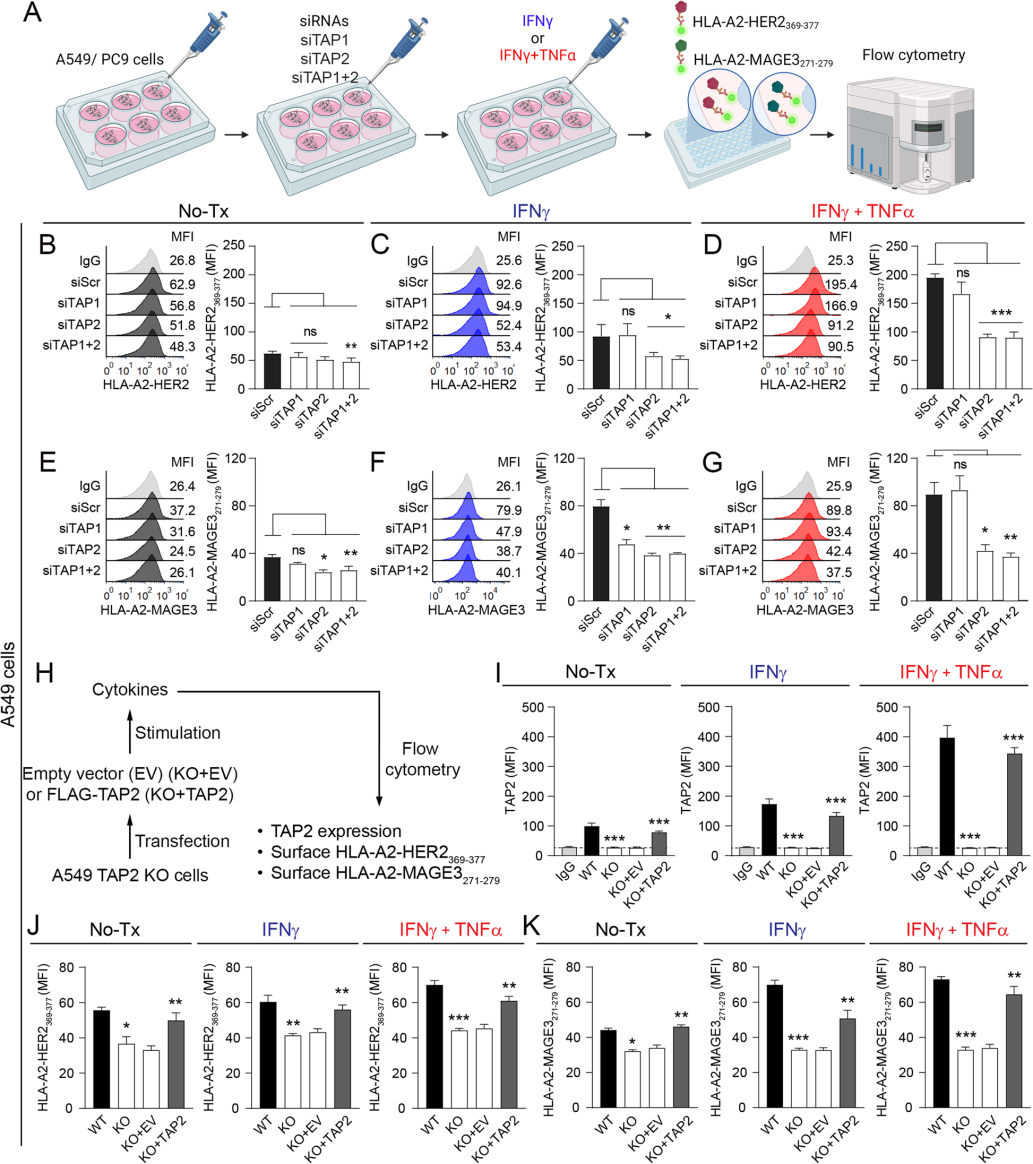
Downregulation of TAP2 reduces the surface levels of peptide-HLA complexes in lung cancer cells. **A** Outline of the experimental strategy used to measure changes in the levels of selected HLA-peptide complexes in lung cancer cells with or without TAP1 and/or TAP2 downregulation using flow cytometry. **B-G** A549 lung cancer cells were transfected with scrambled/control siRNA or with TAP1 and/or TAP2 targeting siRNAs and left untreated (black histograms) or stimulated with IFNγ (blue histograms) or IFNγ + TNFα (red histograms). Panels B-D show the surface levels of HLA-A2-HER2_369-377_, and panels E–G show the levels of HLA-A2-MAGE3_271-279_. **H–K** A549 *TAP2* knockout (KO) cells were transfected with an empty vector (KO + EV) or with a vector containing FLAG-TAP2 (KO + TAP2) and left untreated (black) or stimulated with cytokines IFNγ (blue) or IFNγ + TNFα (red). I, Graphs showing the levels of TAP2 protein analyzed by flow cytometry with or without *TAP2* gene elimination, J-K, Surface levels of HLA-A2-HER2_369-377_ or HLA-A2-MAGE3_271-279_ in A549 cells with or without *TAP2* gene elimination. For panels B-G and I-K, an isotype control antibody (IgG) was used as a background signal reference. Data presented as the mean ± s.d.; *, p < 0.05; **, p < 0.01; ***, p < 0.001 determined by two-tailed unpaired Student’s t-test with a Holm-Bonferroni correction for multiple comparisons. For panels B-G, Scr transfected cells were used as a control for statistical comparison, and for I-K parental wild type (WT) cells were compared with *TAP2* deleted cells (KO) and *TAP2* deleted plus EV (KO + EV) or with *TAP2* deleted with posterior *TAP2* transfection (KO + TAP2). MFI, mean fluorescent intensity; si, siRNA; scr, scrambled; Tx, treatment; ns, not significant

In non-malignant cells, TAP1 and TAP2 form transporters in the ER membrane mediating the active transport of short immunoproteasome-derived peptides into the ER lumen for subsequent processing and loading of high affinity binders onto HLA molecules by the peptide loading complex [4, 26]. The availability of high affinity peptides in the ER is essential for the structural stabilization of the surface HLA-peptide complexes and subsequent delivery to the cell surface for T-cell recognition [27, 28]. Therefore, disruption in TAP1/2 proteins in malignant cells could affect the levels of surface peptide HLA-complexes and reduce the tumor recognition by cognate effector tumor antigen-specific T-cells. To address this, we established a flow cytometry assay to measure specific HLA-peptide complexes expressed in NSCLC cells using the monoclonal antibody (mAb) 4F10 specific to HLA-A2 bound to the HER2 peptide KIFGSLAFL corresponding to amino acids 369–377 of the full-length HER2 protein (HER2_369-377_) [16] or the mAb 12B6 specific to HLA-A2 bound to the MAGE3 nonamer FLWGPRALV corresponding to amino acids 271–279 of the full-length MAGE3 protein (MAGE3_271-279_) [17] (Fig. 2A). Results supporting the specificity of this assay using peptide-HLA loading/unloading experiments with short acid pulses and analysis of HLA-A2 and/or HER2 negative lung cancer cells are discussed in the methods section and shown in the supplementary Fig. S2A-E. We then measured the levels of surface HLA-peptide complexes in two human NSCLC cell lines (e.g. A549 and PC9) with or without targeted TAP1 and/or TAP2 downregulation using a pool containing 4 different small interference RNAs (siRNAs). The siRNAs prominently reduced the baseline *TAP1/2* mRNA expression (~ 90%) and prevented the increase in TAP1/2 protein levels induced by the proinflammatory cytokines IFNγ ± TNFα (Supplementary Fig. S3A-E). As expected for malignant cells, the baseline surface HLA-A2-HER2_369-377_ and HLA-A2-MAGE3_271-279_ complexes levels in control/unmodified A549 cells (KRAS mutant) were relatively low and increased following treatment with proinflammatory cytokines (Figs. 2B-G). TAP1 and/or TAP2 downregulation induced a ~ 5–25% reduction in the levels of peptide-HLA-A2 complexes in unstimulated cells that was more pronounced in cells with TAP2 loss or with concurrent TAP1/TAP2 silencing than in those with reduced TAP1 (Figs. 2B and 2E)_._ In addition, TAP2 downregulation prominently decreased the levels of surface peptide-HLA complexes after treatment with proinflammatory cytokines IFNγ ± TNFα (Figs. 2C-D and 2F-G). TAP1 downregulation alone had a lower impact on the surface antigen levels after cytokine stimulation, and TAP1/TAP2 silencing produced an effect comparable to TAP2 reduction alone supporting a dominant effect of this protein (Figs. 2B-G). Similar results were obtained in the human lung adenocarcinoma cells PC9 (EGFR mutant) displaying higher baseline TAP2 expression than A549 (Supplementary Figs. S5A-F). A similar reduction in the levels of surface HLA-I proteins (HLA-ABC) was seen in TAP1/2 deficient cancer cells, supporting reduced membrane stabilization of HLA-peptide complexes (supplementary Fig. S4A).

Due to the more prominent effect of TAP2 reduction, and to assess the possible contribution of residual TAP2 expression after siRNA-based silencing, we measured the surface peptide-HLA complexes levels in A549 cells with targeted *TAP2* elimination using CRISPR/Cas9-based gene editing with or without posterior-expression of the full-length *TAP2* gene (Fig. 2H). *TAP2* elimination slightly reduced the surface levels of HLA-A2-HER2_369-377_ and HLA-A2-MAGE3_271-279_ in unstimulated A549 cells, but prominently prevented the increase induced by IFNγ ± TNFα (Figs. 2I-K). The re-expression of *TAP2* containing a short amino acid tag located in the N-terminal domain for selective identification (FLAG-TAP2) in *TAP2* knockout cells restored the HLA-A2-HER2_369-377_ and HLA-A2-MAGE3_271-279_ surface expression to a level comparable to parental cells with wild type (endogenous) *TAP2* (Figs. 2I-K and S4B). Together, these results indicate that TAP2 downregulation limits the levels of surface HLA class-I antigens in lung cancer cells under both control and proinflammatory-like conditions.

### TAP2 downregulation protects lung cancer cells from tumor antigen-specific T-cell killing

**Fig. 3:**
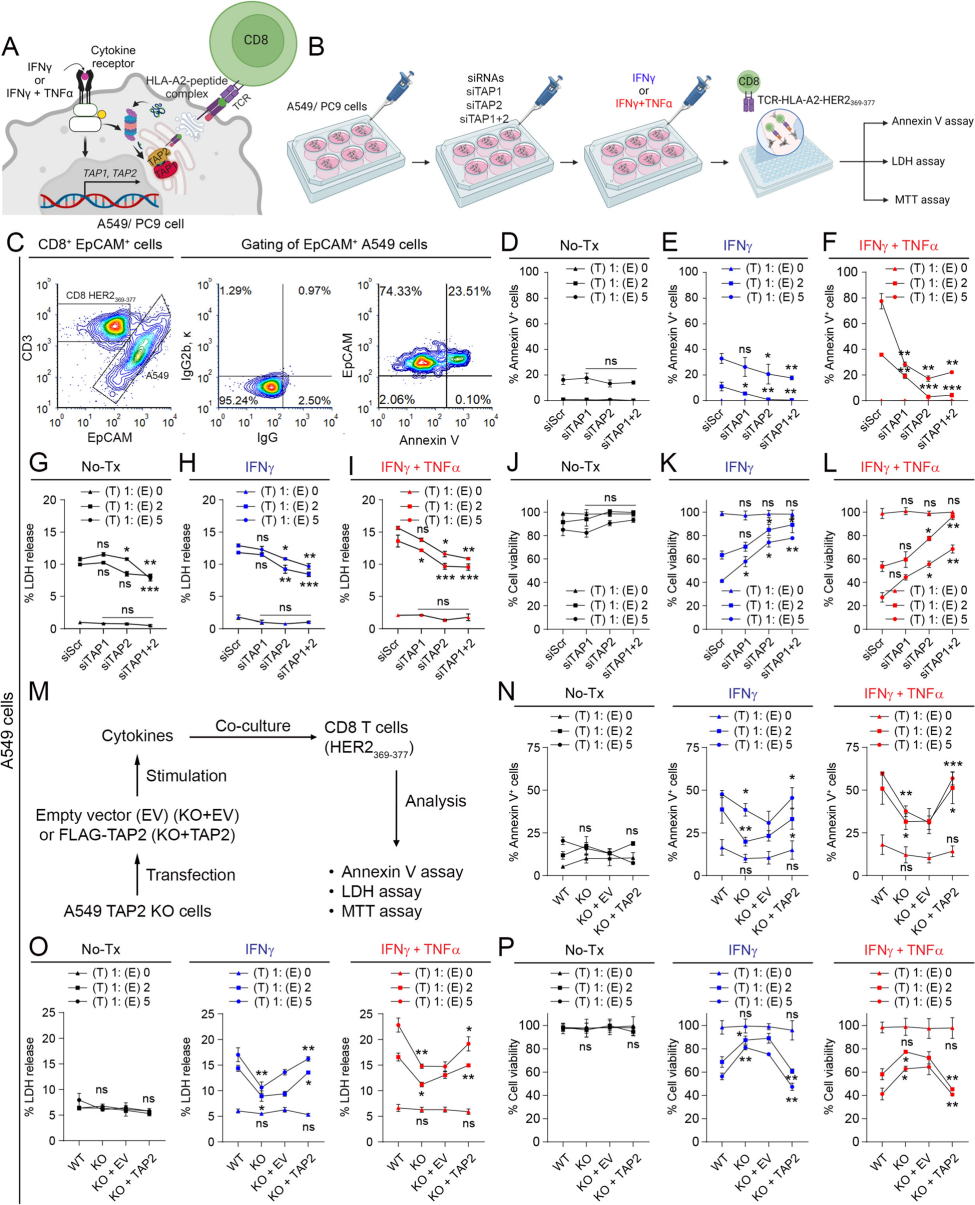
TAP2 downregulation protects cancer cells from tumor antigen-specific CD8 T-cell killing. **A**, **B** Schema and outline of the experimental strategy used to measure tumor antigen-specific killing of lung cancer cells by cognate CD8^+^ T-cells using flow cytometry, LDH release and MTT assay. **C-L** A549 lung cancer cells were transfected with scrambled siRNA or with *TAP1* and/or *TAP2* targeting siRNAs; and left untreated (black) or stimulated with IFNγ (blue) or IFNγ + TNFα (red). After treatment, target lung tumor cells were co-cultured with effector (CD8^+^ T-cells) cells in the ratios of 1:0, 1:2 and 1:5, respectively. Panel C shows the flow cytometry gating strategy to assess cell apoptosis in cancer cell/T-cell co-cultures using the markers CD3 (for CD8^+^ T-cells), EpCAM (for tumor cells) and Annexin V. Panels D-F show the percentage of EpCAM^+^ and Annexin V^+^ apoptotic cancer cells. Panels G-I show the percent of LDH release, and panels J-L represent the cellular viability using MTT assay. **M-P** A549 *TAP2* knockout (KO) cells transfected with empty vector (KO + EV) or FLAG-TAP2 (KO + TAP2) and left untreated or stimulated with IFNγ or IFNγ + TNFα were co-cultured with tumor antigen specific CD8^+^ T-cells at different target cell (tumor): effector (CD8^+^ T-cell) cell ratios. An isotype control antibody (IgG) was used as a background signal reference. Data are presented as the mean ± s.d.; ^*^, p < 0.05; ^**^, p < 0.01; ^***^, p < 0.001 determined by two-tailed unpaired Student’s t-test with a Holm-Bonferroni correction for multiple comparisons. For panels D-L, Scr transfected cells were used as a control for statistical comparison, and for N-P wild type (WT) compared with KO and KO + EV or with KO + TAP2 cells. si, siRNA; scr, scrambled; E, effector CD8 T cells; T, target tumor cells; Tx, treatment; ns, not significant; WT, wild type. See also supplementary Fig. S5-S6

To determine the role of TAP1 and TAP2 in T-cell immune evasion, we co-cultured A549 cells with or without TAP1 and/or TAP2 downregulation, with human HLA-A2^+^/CD8^+^ T-cells expressing the _αβ_TCRs recognizing HLA-A2-HER2_369-377_ (Fig. 3A). Then, we measured cancer cell apoptosis via surface Annexin V staining of EpCAM^+^ malignant cells and cancer cell viability by LDH release and MTT assay in preparations before or after stimulation with proinflammatory cytokines and using different effector (E) to target (T) cell ratios (Figs. 3B-C). Cytokines in these experiments were applied only to cancer cells before co-culturing, and they were removed before adding T-cells to avoid direct lymphocyte stimulation. As shown in Figs. 3D-F, virtually no Annexin V positivity was detected in preparations containing only cancer cells with or without TAP1/2 silencing or after pre-incubation with IFNγ ± TNFα (e.g. (T) 1: (E) 0). Comparable results were seen using the LDH release and MTT assays (Figs. 3G-L). In samples containing both A549 and CD8^+^ T-cells, the downregulation of TAP1 induced no detectable changes in T-cell killing under control/unstimulated condition (Fig. 3D, 3G and 3J), and only partially increased the cancer cell viability in samples pre-incubated with proinflammatory cytokines (Fig. 3E-F, 3H-I and 3K-L). In contrast, TAP2 downregulation prominently increased the viability of unstimulated A549 cells and almost totally prevented the tumor antigen-specific T-cell mediated killing induced by proinflammatory cytokines (Fig. 3D-3L). The concurrent downregulation of both TAP1 and TAP2 produced an effect comparable to TAP2 downregulation alone. Similar results were obtained using PC9 cells as T-cell targets (supplementary Figs. S5G-J), as well as in A549 cells with *TAP2* gene elimination, in which the cancer cell survival increase was suppressed by the re-introduction of *TAP2* (Fig. 3M-P). These results indicate that TAP2 downregulation mediates T-cell immune evasion and protects lung cancer cells from tumor antigen-specific killing.

To assess the impact of TAP2 loss on surface HLA-peptide complexes stabilization and tumor antigen-specific cancer cell killing, we performed peptide re-loading experiments by exposing parental or TAP2-deficient A549 cells to purified HER2_369-377_ or MAGE3_271-279_ peptides. As shown in the supplementary Fig. S6A, the exogenous addition of the 9-mers only slightly increased the levels of surface HLA-peptide complexes in unmodified A549 cells, but prominently increased the detection in TAP2-deficient cells. In addition, reloading of the HER2_369-377_ peptide prominently increased the tumor-antigen specific killing by HLA-A2-HER2_369-377_ specific T-cells in TAP2-deficient cancer cells with minimal effect in parental controls or in TAP2-deficient cells exposed to MAGE3_271-279_ (Supplementary Fig. S6B-C). Together, these results indicate a prominent role of HLA-peptide complexes in tumor-antigen specific T-cell recognition and killing. They also suggest the presence of surface HLA-peptide complexes loaded with lower affinity binders in TAP2-deficient cells than in parental cells that can be rapidly/effectively displaced by competing higher affinity exogenous nonamer peptides, as previously reported [29, 30].

### TAP2 downregulation suppresses proinflammatory responses of lung cancer cells via SOCS1 upregulation

**Fig. 4:**
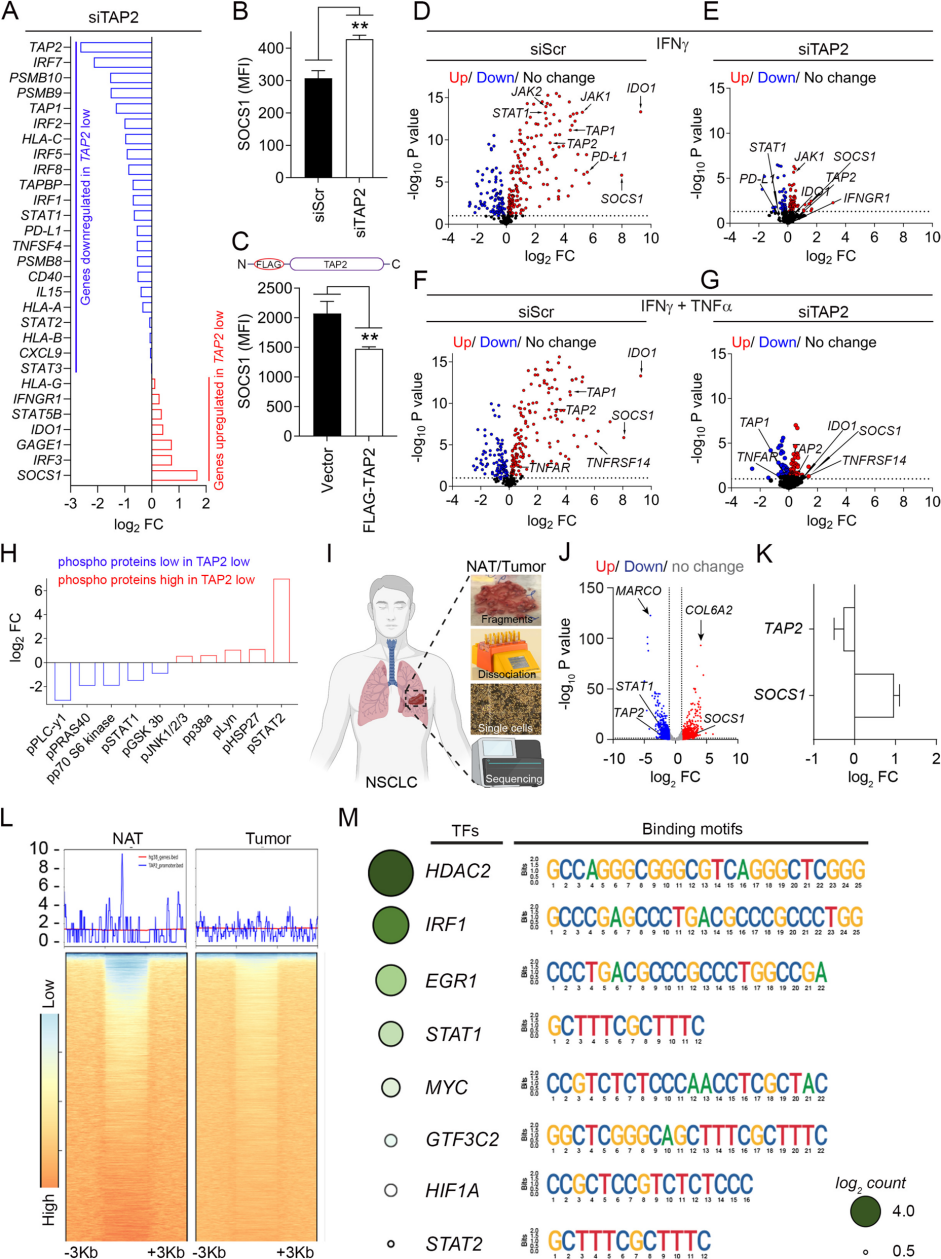
Downregulation of TAP2 alters intracellular immunomodulatory pathways via SOCS1 upregulation and TAP2 downregulation in human NSCLC is due to epigenetic changes. **A** A549 cells were transfected with scrambled siRNA or with TAP2 targeting siRNAs followed by targeted transcriptomic analysis. Graph shows differentially expressed genes in *TAP2* silenced versus control cells ranked based on low (blue) to high (red) transcript expression. **B**, **C** SOCS1 protein expression by flow cytometry in A549 cells. B, Cells were transfected with scrambled siRNA or with *TAP2* targeting siRNAs C, Cells were transfected with empty vector or with the full-length TAP2 including a FLAG octapeptide. **D-G,** A549 cells were transfected with TAP1/2 siRNAs as indicated in **3A** and stimulated with IFNγ (**D**, **E**) or with IFNγ plus TNFα (**F**, **G**). Volcano plots representing differential expression of IFNγ pathway signature genes. **H** A549 cells were transfected with TAP1/2 siRNAs followed by stimulation with IFNγ plus TNFα. Phosphoprotein levels were measured and ranked based on low (blue) to high (red) expression in *TAP2* silenced vs control cells. **I-M** Normal adjacent to tumor (NAT) and lung tumor tissues surgically resected from NSCLC patients with low TAP2 protein levels. **I** Schematic of the strategy for obtaining and analyzing single cell preparations. **J** Volcano plot showing the differential gene expression of paired lung cancer relative to NAT using RNA sequencing. **K** Fold change of the mRNA expression of *TAP2* and *SOCS1* in tumor samples relative to NAT, **L** Heatmap of ATAC-seq analysis representing chromatin accessibility in the TAP2 promoter region. **M** Predicted transcription factor (TF) binding sites with the highest affinity scores (log2 count) for the *TAP2* promoter region. Data are presented as the mean ± s.d. ^**^, p < 0.01 determined by two-tailed unpaired Student’s t-test. FC, fold change; MFI, mean fluorescent intensity; si, siRNA; scr, scrambled; TFs, transcription factors. See also supplementary Fig. S7

Because of the marked impact of *TAP2* silencing in peptide-HLA complexes and T-cell evasion after treatment with IFNγ ± TNFα, we hypothesized a role of TAP2 in the control of proinflammatory signals and/or cytokine sensitivity. To assess this, we first compared the immune transcriptomic profiles of control and TAP2-downregulated A549 cells in the absence of T-cells or cytokine stimulation. As shown in Fig. 4A, downregulation of *TAP2* prominently reduced the expression of TNF superfamily genes (e.g., *TNFSF4*), HLA class-I APM transcripts (e.g. *HLA-A, -B, -C, TAP1, TAP2, TAPBP, PSMB8, PSMB9* and *PSMB10*) and intracellular signaling regulators (e.g. *STAT1, STAT2, IRF1*, *STAT6* and *CD40*). *TAP2* downregulation was also associated with reduced expression of chemokines involved in T- and NK-cell recruitment and activation including *CXCL9* and *IL-15* [31, 32]. No reduction was seen in the levels of the IFNγ receptor.

*IFNGR1* or the TNFα receptors *TNFRSF1A* and *TNFRSF1B*. Analysis of transcripts significantly upregulated in *TAP2* deficient cells identified *SOCS1* (suppressor of cytokine signaling 1) as the top increased gene and this molecule has been previously shown in multiple studies to effectively suppress the IFNγ/STAT pathway and TNFα signaling [33, 34]. SOCS1 protein was also significantly higher in A549 cells after TAP2 downregulation (Fig. 4B) and the expression of TAP2 reduced SOCS1 protein levels in lung cancer cells supporting a bi-directional modulation (Fig. 4C and Supplementary Fig. S7). *TAP2* silencing also reduced the IFNγ-mediated increase in multiple proinflammatory transcripts including *STAT1, JAK1*, *JAK2*, and *TAP1*, as well as immunosuppressive IFNγ-inducible genes such as *JAK1, PD-L1* and *IDO-1* (Figs. 4D and 4E). *TAP2* downregulation similarly prevented the increase of multiple additional proinflammatory transcripts including *TNFRA* and *TNFRSF14* after treatment with combined IFNγ + TNFα (Figs. 4F-G). By phosphoprotein array analysis, *TAP2* silencing markedly reduced the levels of multiple proinflammatory and pro-mitogenic/migration signals in response to IFNγ + TNF including pPLC-γ1, pRAS40, pp70S6k, pSTAT1, and pGSK3b. *TAP2* downregulation also prominently increased the levels of pSTAT2 associated with both proinflammatory and immune suppressive effects (Fig. 4H) [35, 36]. Together, these results show that TAP2 expression can modify key intracellular signaling pathways and limit the response to proinflammatory cytokines in lung cancer cells.

### TAP2 downregulation in NSCLC is due to epigenetic modifications

To investigate mechanisms mediating cancer cell TAP2 downregulation in NSCLC, we evaluated the frequency of deleterious *TAP2* genetic variants in human lung adenocarcinomas and squamous-cell carcinomas from The Cancer Genome Atlas (TCGA) cohort analyzed using whole exome DNA sequencing. As shown in the supplementary Fig. S8A, only 13 (1.5%) of 875 individual NSCLC cases contained deleterious mutations of the *TAP2* gene that could explain the protein downregulation, supporting a non-genomic mechanism of TAP2 silencing. The analysis of fresh single cell suspensions from primary resected NSCLCs with TAP2 downregulation and adjacent morphologically normal lung tissue from the same patients using mRNA sequencing confirmed the association between low *TAP2* and elevated *SOCS1* expression in human malignancies and suggested a transcriptional mechanism underlying TAP2 downregulation (Figs. 4I-K). Analysis of these samples using ATAC-seq revealed reduced chromatin accessibility in the *TAP2* gene promoter region spanning ± 3 kb around the transcription starting site in NSCLC as compared to non-tumor lung tissue, supporting epigenetic silencing of TAP2 (Fig. 4L). The sequence analysis of the promoter sites with differential accessibility in malignant vs nonmalignant samples identified numerous consensus motifs for binding of transcription factors involved in proinflammatory responses including *HDAC2*, *IRF1*, *EGR1*, *STAT1*, *MYC*, *GTF3C2*, *HIF1A* and *STAT2* (Fig. 4M).

### Tumor-derived IL-4 reduces TAP2 and mediates T-cell evasion in lung cancer

**Fig. 5:**
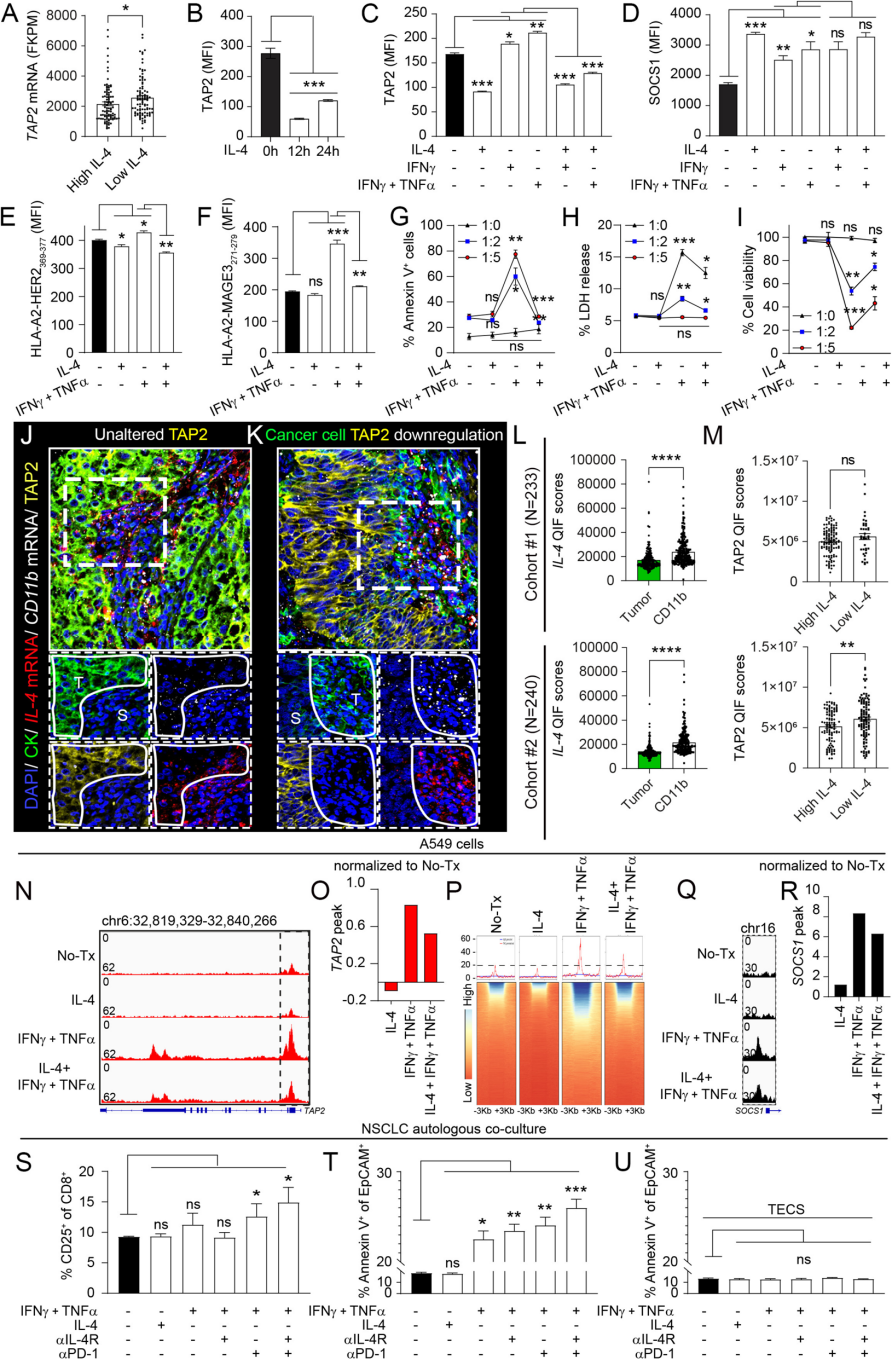
Myeloid cell-derived IL-4 reduces TAP2 expression via epigenetic remodeling in lung cancer. **A** TCGA NSCLC cohort analysis of *TAP2* expression stratified by the median *IL-4* mRNA levels. **B** A549 cells were treated with IL-4 for 0-24 h and TAP2 protein levels were measured using flow cytometry. **C-I** A549 cells were treated with IL-4, IL-4 + IFNγ or IL-4 with IFNγ + TNFα. C, TAP2 protein levels measured by flow cytometry, D, SOCS1 protein levels by flow cytometry, E, Surface levels of HLA-A2-HER2_369-377_ complexes and F, HLA-A2-MAGE3_271-279_ complexes measured by flow cytometry. **G-I** A549 cells (target) were treated with IL-4 ± IFNγ + TNFα and incubated with effector tumor antigen-specific CD8^+^ T-cells to measure, G, cancer cell killing by Annexin V positivity, H, LDH release and I, cellular viability using MTT assay. **J-M** Fluorescence images and signal measurement from multiplexed spatial analysis of protein and mRNA transcripts in NSCLCs from Cohorts #1 and #2. J-K, Representative captions of cytokeratin (CK, green), *IL-4* mRNA (red), *CD11b* mRNA (white), TAP2 protein (yellow) and nuclei (blue). L, expression levels of *IL-4* mRNA measured selectively in CK^+^ tumor cells or in *CD11b*^+^ myeloid cells. M, expression levels of TAP2 protein in CK^+^ tumor cells stratified by the median *IL-4* mRNA expressed in CD11b^+^ myeloid cells. **N-R** A549 cells were treated with IL-4 ± IFNγ + TNFα and analyzed using ATAC-seq. N, ATAC-seq promoter peak enrichment values of the *TAP2* gene. O, comparative analysis of the *TAP2* ATAC-seq promoter peak enrichment after different cytokine treatments in A549 cells. P, heatmap of ATAC-seq analysis representing chromatin accessibility in the *TAP2* promoter region after cytokine treatments. Q, promoter peak enrichment values of the *SOCS1* gene. R, comparative analysis of the *SOCS1* ATAC-seq promoter peak enrichment after cytokine treatments. Promoter regions were considered as the DNA sequences between the gene Transcription Start Site (TSS, + 3 kb) and Transcription End Site (TES, -3 kb). **S-U** Autologous single cell suspension cultures including cancer and immune cells from primary NSCLC tissues stimulated with IFNγ + TNFα followed by incubation with IL-4Rα (αIL-4R) or PD-1 blocking antibodies (αPD-1). S, levels of CD8^+^/CD25^+^ T-cells measured by flow cytometry. T, percentage of EpCAM^+^/Annexin V^+^ apoptotic cancer cells in autologous cell suspensions. U, percentage of EpCAM^+^/Annexin V^+^ apoptotic cancer cells in autologous cell suspensions with selective elimination of CD3^+^ T-cells (TECS). Isotype (IgG) was used as a background signal control. Data are presented as the mean ± s.d. *, p < 0.05; **, p < 0.01; ***, p < 0.001; ****, p < 0.0001 determined by two-tailed unpaired Student’s t-test with a Holm-Bonferroni correction for multiple comparisons. B-I and S-U, untreated cells were used as a control. E, effector CD8^+^ T-cells; FPKM, fragments per kilobase million; ns, not significant; MFI, mean fluorescent intensity; T, tumor; S, stroma. See also supplementary Figs. S8-S12

We hypothesized that accumulation of immune suppressive cytokines in the tumor microenvironment could participate in the epigenetic *TAP2* silencing on malignant cells. Exploration of the level of multiple cytokines and *TAP2* transcripts in the TCGA NSCLC datasets confirmed the expected positive association between elevated *IFNγ* and *TAP2* (Supplementary Fig. S8B). In the same cohort, high expression of *IL-4* mRNA, but not of other immunosuppressive cytokines such as *IL-8*, was significantly associated with lower *TAP2* expression, suggesting a negative modulatory effect of IL-4 on TAP2 levels (Fig. 5A and supplementary Fig. S8C). Consistent with this notion, in vitro treatment of A549 cells with human IL-4 for 12-24 h prominently reduced the TAP2 protein levels (Fig. 5B) and *TAP2* mRNA expression (Supplementary Fig. S8D). In addition, exposure of lung cancer cells to IL-4 suppressed the increase in TAP2 protein induced by IFNγ or IFNγ + TNFα (Fig. 5C). Treatment with IL-4 also increased the levels of SOCS1 protein in A549 cells, but did not affect the SOCS1 increase induced by proinflammatory cytokines (Fig. 5D). Like TAP2 downregulation, IL-4 only slightly decreased the baseline levels of surface peptide-HLA complexes, but almost totally prevented the increase in surface HLA-A2-HER2_369-377_ or HLA-A2-MAGE3_271-279_ after treatment with proinflammatory cytokines (Figs. 5E-F). Comparable responses in TAP2, SOCS1 levels and surface peptide-HLA complexes after IL-4 treatment were observed in PC9 lung cancer cells (Supplementary Figs. S10A-E). The *IL-4* mRNA levels in the TCGA lung cancer dataset were not significantly associated with *TAP1* expression and treatment with IL-4 did not significantly affect the TAP1 protein levels in lung cancer cells (Supplementary Figs. S9A-B).

To further determine the role of IL-4 in adaptive immune evasion, we studied cancer cell killing in co-cultures of A549 cells with tumor antigen-specific CD8^+^ T-cells with or without exposure to IL-4 and/or IFNγ + TNFα. As shown in Figs. 5G-I, IL-4 alone only slightly reduced the T-cell killing in control/unstimulated A549 cells, but almost totally suppressed the tumor antigen-specific killing induced by proinflammatory cytokines. IL-4 treatment of *TAP2* knockout A549 cells only slightly affected the levels of surface HLA-A2-HER2_369-377_ or HLA-A2-MAGE3_271-279_ peptide complexes and the CD8^+^ T-cell killing, and produced a comparable effect in parental and in *TAP2* knockout cancer cells with *TAP2* re-expression (Supplementary Figs. S9C-H). Collectively, these results reveal that IL-4 reduces TAP2 expression, mimics the effects of TAP2 downregulation and mediates adaptive immune evasion of human lung cancer cells. These results also show that the defects in TAP2 induced by IL-4 are not reverted or blocked by treatment with proinflammatory cytokines.

### IL-4 is produced by intratumor myeloid cells and blockade of the IL-4 pathway restores T-cell killing of lung cancer cells

To identify the cellular source of IL-4, we spatially mapped the *IL-4* mRNA production in the tumor microenvironment of human NSCLCs using QIF. We prioritized the in situ analysis of *IL-4* mRNA over IL-4 protein to minimize the possible (differential) impact of pre-analytical variables such as warm/cold ischemia and fixation time in IL-4 secretion across cases. We simultaneously analyzed the expression of TAP2 protein, *IL-4* mRNA, *CD11b* mRNA and CK protein in tumor specimens from the NSCLC Cohorts #1 and #2. As shown in Figs. 5J-K, cancer cell TAP2 protein downregulation was spatially associated with high local *IL-4* mRNA expression. The *IL-4* mRNA transcripts were detected both in *CD11b*-expressing intratumor myeloid cells and focally in CK-positive epithelial malignant cells. Quantification of the markers within specific cell type compartments revealed higher *IL-4* mRNA expression in intratumor myeloid cells than in cancer cells, as well as lower TAP2 protein levels in cases with high myeloid cell *IL-4* mRNA expression (Figs. 5L-M). Notably, the majority of *IL-4*^+^/*CD11b*^+^ myeloid cells were located within the cancer cell nests and not in the CK-negative stromal tissue compartment (Supplementary Figs. S11A-B).

To determine the mechanism mediating TAP2 reduction by IL-4, we analyzed the chromatin changes by ATAC-seq of A549 cells with or without treatment with IL-4 and/or proinflammatory cytokines. As shown in Fig. 5N control/untreated A549 cells showed a single chromatin accessibility peak located in the *TAP2* promoter region. Exposure to IL-4 markedly reduced the accessibility peak in this region (~ 50% reduction vs control), and treatment with IFNγ + TNFα increased the chromatin accessibility of the *TAP2* gene promoter and of intragenic regions located in exon 12. The concurrent treatment with IL-4 and IFNγ + TNFα prominently reduced the peak signals induced by proinflammatory cytokines alone and the effect was comparable in both the promoter and the intragenic peak (Figs. 5N-P). Treatment with IL-4 also increased the chromatin accessibility of the *SOCS1* gene promoter region under both control conditions and after treatment with IFNγ + TNFα (Figs. 5Q-R).

To further assess the role of the IL-4 pathway in TAP2 mediated cancer cell immune evasion and immunotherapy resistance, we studied the role of the IL-4 receptor α (IL-4Rα) blockade in cancer cell elimination using primary autologous tumor/immune-cell co-cultures obtained from disaggregated human NSCLCs expressing low TAP2 levels (Figs. 5S-U and supplementary Figs. S12A-C). Pre-incubation of cancer cells with IL-4Rα blocking antibodies alone did not significantly affect control/unstimulated cell preparations. In addition, and as shown in Fig. 5S, concurrent treatment of malignant cells with proinflammatory cytokines and IL-4Rα blockade only slightly affected CD8^+^ T-cell activation as measured by surface CD25 levels. Exposure of the tumor/immune cell co-cultures to the PD-1 blocking monoclonal antibody Pembrolizumab significantly enhanced the effector T-cell activation induced by proinflammatory cytokines and the concurrent blockade of PD-1 and IL-4Rα induced higher CD8^+^ T-cell activation. In addition, IL-4Rα blockade in the presence of IFNγ + TNFα significantly enhanced the autologous T-cell mediated cancer cell killing as measured by Annexin V positivity of EpCAM-expressing lung cancer cells and promoted the cancer cell elimination induced by PD-1 blockade (Fig. 5T). These responses were absent in primary tumor/immune cell co-cultures with elimination of T-cells using negative selection with CD3-containing magnetic beads (e.g. CD3 T-cell eliminated cell suspension or TECS), evidencing that the effect of treatments was solely mediated by T-cells (Fig. 5U). Together, these results indicate that IL-4 production by intratumor myeloid cells can reduce TAP2 expression and mediate immunotherapy resistance in human NSCLC.

### TAP2 downregulation in lung cancer cells is reversible and can be increased using available compounds

**Fig. 6:**
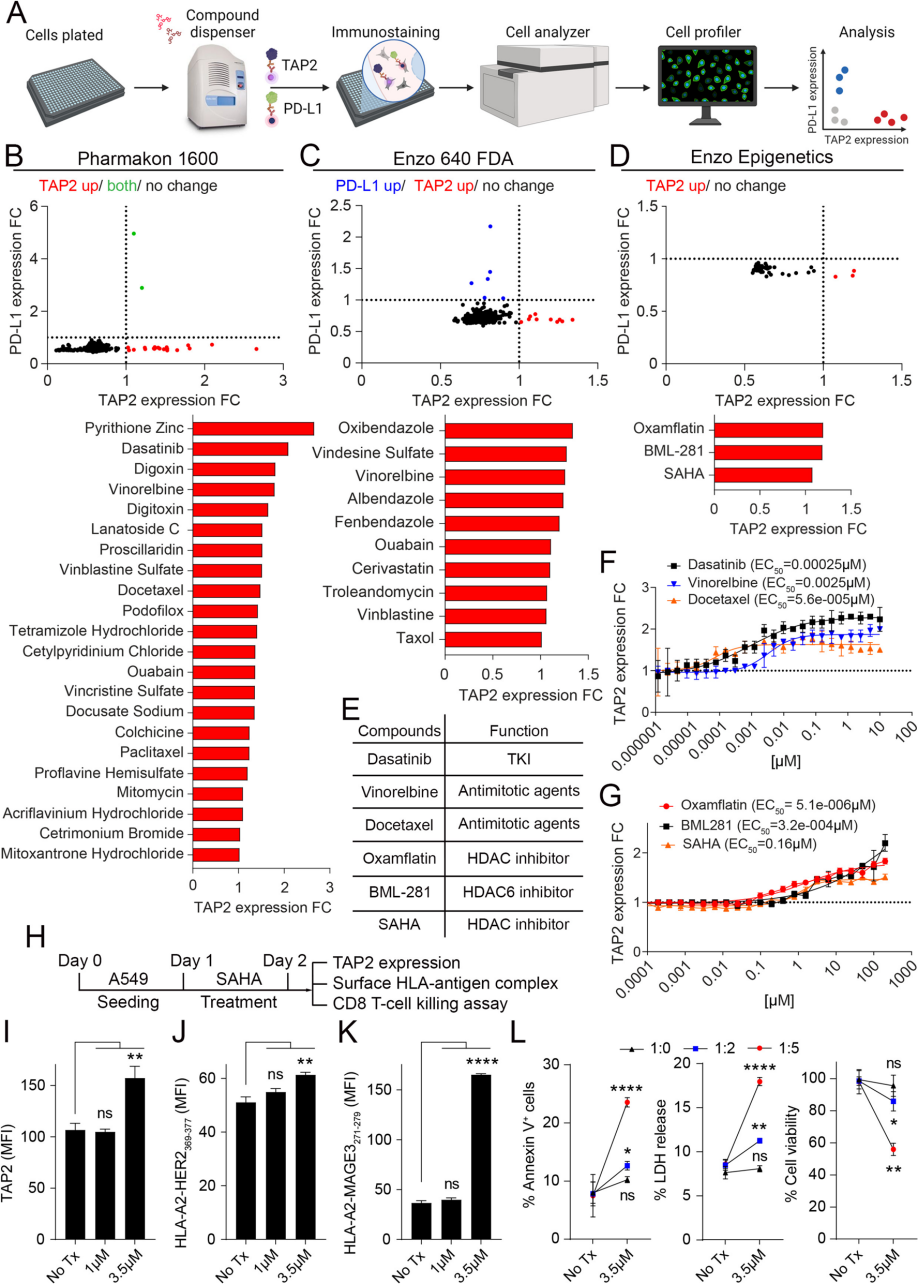
Restoration of TAP2 protein expression induces tumor cell surface antigenicity in lung cancer cells. **A** Schematic showing the strategy for high throughput screening of pharmacologic agents using TAP2 and PD-L1 immunostaining in A549 cells. **B-D** Representative plots showing the upregulation of TAP2 protein (red) or PD-L1 protein (blue) or both (green) in A549 cells treated with individual compounds from the Pharmakon 1600 library (B), Enzo 640 FDA library (C) and Enzo epigenetic library (D). The scores for each compound were calculated as TAP2 protein level fold change and normalized to the vehicle control (DMSO) treated group. **E** Summary of TAP2 protein inducer compounds selected based on representation of all 3 libraries. **F-G** Dose–response curves to determine EC_50_ values for TAP2 protein upregulation in A549 cells using selected compounds shown in E. **H** Experimental outline for SAHA/Vorinostat treatment and analysis of A549 cells. **I** Levels of TAP2 protein measured by flow cytometry. **J** surface HLA-A2-HER2_369-377_ complexes and **K** surface HLA-A2-MAGE3_271-279_ levels measured by flow cytometry. **L** Levels of apoptotic cancer cell death (Annexin V staining) and cell viability (LDH release and MTT assay) of parental A549 cells co-incubated with tumor antigen-specific effector CD8^+^ T-cells using different effector to target cell ratios (1:0, 1:2 and 1:5) with or without treatment with 3.5 µM SAHA. An isotype control antibody (IgG) was used as a background signal reference. Data are presented as the mean ± s.d.; *p < 0.05; **p < 0.01; ****, p < 0.0001 determined by two-tailed unpaired Student’s t-test. FC, fold change; ns, non-significant; No-Tx, no treatment; MFI, mean fluorescent intensity; TKI, tyrosine kinase inhibitor. See also supplementary Fig. S13

To identify compounds able to restore TAP2, we performed a high throughput pharmacologic screen including 2,283 individual compounds in 3 libraries to identify agents able to increase TAP2 protein levels in A549 cells. The screen included 2 generic libraries of 2,240 FDA approved drugs (Pharmacon 1600 and Enzo 640 FDA) and 1 library including 43 epigenetic modulators. All the compounds were initially screened using a concentration of 10 μM and the effect of the compounds was evaluated individually (e.g. single agent) and based on their capacity to increase TAP2 protein expression by direct immunofluorescence using automated signal quantification (Fig. 6A). In addition, the screen included as a second parameter the changes in PD-L1 protein levels, with the goal of selecting agents able to restore TAP2 without increasing PD-L1 mediated tumor immune evasion. As shown in Figs. 6B-D, each library identified positive hits with a TAP2 protein fold increase > 1 relative to control (DMSO as vehicle control) cells. The compounds identified in the FDA approved drug libraries included several antineoplastic agents such as Dasatinib, Vinorelbine, Docetaxel, Vinblastine, Vindesine and Mitomycin; the anti-inflammatory/anti-gout agent Colchicine, the cardiac glycosides Digoxin and Ouabain; as well as the anti-parasitic/anti-fungal agents Pyrithione Zinc, Oxibendazole, Albendazole, Fenbendazole and Podofilox (Figs. 6B-C). Notably, multiple compounds were identified independently in separate libraries, supporting the consistency of the findings. Three of the compounds identified in the FDA approved libraries were previously shown to have anti-tumor activity in NSCLC patients alone or in combination with immunotherapy including Dasatinib, Vinorelbine and Docetaxel (Fig. 6E). The epigenetic library identified 3 compounds able to increase TAP2 expression including the non-selective HDAC inhibitor Oxamflatin, the HDAC6 inhibitor BML-281, and the class-I/II HDAC inhibitor SAHA/Vorinostat (Figs. 6D-E).

To explore the potency and dose–effect response of these compounds on TAP2 levels, we expanded the studies to test multiple concentrations and determine the half maximal effective concentration (EC_50_) of 6 compounds selected from the libraries including Dasatinib, Vinorelbine, Docetaxel, Oxamflatin, BML-281 and SAHA (Fig. 6E). As shown in Figs. 6F-G, the compound with the highest potency was Docetaxel with an EC_50_ of 0.000056 μM followed by Dasatinib with an EC_50_ of 0.00025 μM and Vinorelbine with 0.0025 μM. The most potent epigenetic modulator was Oxamflatin with an EC_50_ of 0.0000051 μM followed by BML-281 with 0.00032 μM and SAHA/Vorinostat with 0.16 μM. Furthermore, treatment of A549 cells with the FDA approved epigenetic modulator SAHA/Vorinostat increased TAP2 protein expression by flow cytometry (Figs. 6H-I) and enhanced the levels of HLA-A2-HER2_369-377_ and HLA-A2-MAGE3_271-279_ complexes in a concentration-dependent manner (Figs. 6J-K). To further assess the role of HDAC inhibitors in TAP2-mediated adaptive immune evasion, we investigated the cancer cell killing in co-cultures of A549 cells with tumor antigen-specific CD8^+^ T-cells with or without treatment with SAHA. As shown in Fig. 6L, SAHA enhanced T-cell mediated cytotoxicity of A549 cells. Similar results were observed using two selective HDAC6 inhibitors, BML281 and WT161 in both A549 and PC9 lung cancer cells (Supplementary Figs. S13A-P). Collectively, these results reveal the potential for increasing or restoring TAP2 expression in human lung cancer cells and suggest the possibility to repurpose available pharmacologic agents to treat patients with TAP2-deficient NSCLC.

## Discussion

Our results identify cancer cell selective TAP2 downregulation as a dominant, reversible, and potentially actionable mechanism of adaptive immune evasion and immunotherapy resistance in NSCLC. Our studies also reveal a complex and previously unrecognized mechanism underlying TAP2 downregulation in the protection of lung cancer cells to T-cell mediated killing that involves: *i)* limiting the levels of high affinity surface class-I HLA-peptide complexes, *ii)* suppression of proinflammatory signals via SOCS1 upregulation; and *iii)* reduced sensitivity to proinflammatory cytokines. We also identify local IL-4 produced by intratumor myeloid cells as a critical factor inducing TAP2 downregulation in cancer cells and mediating reduced sensitivity to PD-1 axis blockers (Supplementary Fig. S14). These results describe novel functions of TAP2 protein beyond antigen presentation and have prominent biological implications. Our results can also guide the design of biomarker strategies and therapeutic avenues aiming to restore TAP2 expression via IL-4 pathway blockade or through alternative mechanisms such as selective targeting of IL-4 producing intratumor myeloid cells and/or use of pharmacologic compounds.

The spatially resolved analysis by QIF revealed that ~ 42.4% of 819 immunotherapy naive primary NSCLCs from multiple independent cohorts, and including early stage/resectable tumors, show cancer cell selective downregulation of TAP2 protein. A substantial fraction of these cases also displays concurrent TAP1 downregulation. Previous studies have also identified HLA class-I APM component alterations in human solid tumors, including NSCLC [37, 38]. The fraction of cases displaying TAP1 and TAP2 alterations in our study is higher than previous studies in NSCLC ranging from 2.1–7.9% and 18.7–28.78%, respectively using genomic/transcriptomics and chromogenic immunohistochemistry. Differences in the approach, including dissimilar sensitivity of the assay used for the TAP1/2 loss identification could partially explain these differences. Consistent with our results, previous studies have also shown that the alterations in TAP1 and TAP2 proteins in cancer cells are uncommonly associated with deleterious genomic alterations and were suggested to occur as a consequence of protein dysregulation [3]. The lack of structural alterations in the *TAP1* and *TAP2* genes in most human NSCLCs support the possibility of restoring their expression and function using pharmacologic interventions.

One notable aspect of our results is the prominent difference in the frequency of TAP1 and TAP2 cancer cell downregulation in NSCLC, as well as their dissimilar impact on surface antigenic peptide abundance and role in protection of cancer cells from T-cell attack. Although previous studies conducted in non-tumor cells consider the TAP transporter to be a heteromeric structure with both TAP1 and TAP2 proteins equally represented and required for adequate function, an earlier study showed that newly synthesized TAP2 proteins are highly unstable and require physical association with TAP1 to prevent its proteasomal degradation [39]. In addition, chemical cross-linking studies indicated that TAP2, but not TAP1, can form a homodimer complexes both in whole cells and in detergent lysates [40]. A recent study using computational models showed that in the TAP1/TAP2 heterodimeric complex, four residues (M196, R380, M413, and E417) of TAP2 protein have high affinity to interact with HLA class-I type peptides, compared to only one residue (K483) on TAP1 protein, suggesting that TAP2 has a stronger peptide binding function [41]. Together, these results suggest that TAP2 downregulation in cancer cells could be more energy-efficient and produce a deeper immune evasion than TAP1 reduction. Interestingly, our transcriptomic analysis revealed that TAP1 expression is reduced after TAP2 silencing in lung cancer cells, and TAP2 is also reduced after TAP1 downregulation, supporting interdependency in the expression of these proteins.

As expected from its known function in transporting relatively short 8–16 amino acid long peptides into the ER for HLA loading and subsequent surface membrane transport [42], TAP2 downregulation in lung cancer cells reduces the levels of immunogenic surface HLA-peptide complexes expected to limit the recognition and killing by tumor-antigen specific T-cells under proinflammatory conditions. In addition, silencing of *TAP2* reduces the expression of other APM genes and intracellular proinflammatory signaling molecules, and suppresses the expression of potent T-cell/NK cells chemoattractants/activators such as *CXCL9* and *IL-15* [31, 32]. These effects are associated with marked upregulation of SOCS1, a well-established inhibitor of the IFNγ/STAT proinflammatory pathway involved in multiple human diseases [33, 43], and could explain lower adaptive anti-tumor responses and reduced CD8^+^ TILs in TAP2-deficient NSCLCs. The silencing of TAP2 in malignant cells also alters the sensitivity to IFNγ and TNFα, also favoring immune evasion. Together, these results indicate a role of TAP2 downregulation beyond the HLA class-I APM.

Previous studies have shown that IL-4 secretion by cancer cells can have pro-tumor effects mediated by autocrine signaling and suppression of cell death [44, 45], or by induction of alternative polarization of immunosuppressive tumor associated macrophages (TAMs) [46] and monocyte derived suppressor cells (MDSCs) in the tumor microenvironment [47]. To our knowledge, our study is the first to mechanistically link local IL-4 production in the tumor bed with TAP2 reduction, immune evasion, and immunotherapy resistance in cancer. Notably, most of the *IL-4* mRNA production in human NSCLCs is in CD11b-expressing intratumor myeloid cells supporting a multicellular mechanism involving IL-4 production by alternatively polarized TAMs/MDSCs signaling in an autocrine fashion to induce and/or maintain an immune suppressive state of these cells; and paracrine IL-4 signaling on cancer cells reducing TAP2 expression and favoring tumor immune evasion. This is consistent with previous studies showing a prominent role of MDSCs in lung cancer development [48], the upregulation/overexpression of IL-4 receptors in lung cancer cells [49], and the immunostimulatory and anti-tumor effect of IL-4 blockade using monoclonal antibodies in pre-clinical cancer models and recently also in clinical trials [50, 51]. It is envisioned that IL-4 signaling can also induce effects beyond TAP2 downregulation in lung cancer cells.

Available pharmacologic agents can increase TAP2 expression in lung cancer cells and could potentially be tested in therapeutic clinical trials alone or in combination with other agents to treat patients with TAP2-deficient malignancies. It is noticeable that some of these compounds were previously approved to treat patients with advanced NSCLC and can have prominent anti-tumor effects such as Paclitaxel, Docetaxel and Dasatinib. The possible contribution of TAP2 increase in their anti-tumor mechanism of action could explain, at least partially, the marked immunostimulatory effect of some of these treatments [52]. Other compounds identified in our screens could also eventually be repurposed and used in patients with TAP2-deficient NSCLC, if they are able to provide an adequate therapeutic index. Consistent with an epigenetic mechanism mediating TAP2 reduction in lung cancer cells, we identified 4 HDAC inhibitors able to increase TAP2 expression without increasing PD-L1 expression. Additional work will be required to identify novel and more specific compounds able to selectively modulate TAP2 and potentially exert controlled immunomodulatory effects for patients with cancer or autoimmune diseases (e.g. compounds that could selectively reduce TAP2 expression).

We acknowledge the limitations of our work including the use of tissue microarrays to study NSCLC patient cohorts that examine relatively small tissue areas and can over- or underrepresent the tested markers, and the inclusion of retrospective cases that were collected at different time-points and treated in a non-uniform fashion. However, we and others have validated this approach to examine the associations between marker(s) expression, clinicopathological characteristics of tumors and survival in patients with lung cancer [9, 53]. The consistent results obtained across multiple independent cohorts also support our findings. Another limitation is the unavailability of detailed genomic analysis of possible *TAP2* gene alterations in human NSCLCs included in the study as possible contributors to TAP2 downregulation. We also acknowledge limitations in the determination of cancer cell TAP1/2 protein downregulation using QIF due to the lack of a well established biological references to determine relevant cut-points in human tumor specimens.

## Conclusions

Our work establishes the IL-4 mediated cancer cell TAP2 downregulation as a novel, dominant and potentially actionable mechanism of T-cell immune evasion and immunotherapy resistance in human NSCLC and elucidates the mechanism mediating these responses with broad biological and potential clinical implications.

## Supplementary Information

Supplementary Material 1.

## Data Availability

No datasets were generated or analysed during the current study.

## References

[1] Schalper KA, Kaftan E, Herbst RS. Predictive Biomarkers for PD-1 Axis Therapies: The Hidden Treasure or a Call for Research. Clin Cancer Res. 2016;22:2102–4. 26957559 10.1158/1078-0432.CCR-16-0169 PMC4940186

[2] Jhunjhunwala S, Hammer C, Delamarre L. Antigen presentation in cancer: insights into tumour immunogenicity and immune evasion. Nat Rev Cancer. 2021;21:298–312. 33750922 10.1038/s41568-021-00339-z

[3] Thompson JC, Davis C, Deshpande C, Hwang WT, Jeffries S, Huang A, Mitchell TC, Langer CJ, Albelda SM. Gene signature of antigen processing and presentation machinery predicts response to checkpoint blockade in non-small cell lung cancer (NSCLC) and melanoma. J Immunother Cancer. 2020;8:e000974. 33028693 10.1136/jitc-2020-000974 PMC7542663

[4] Sadagopan A, Michelakos T, Boyiadzis G, Ferrone C, Ferrone S. Human Leukocyte Antigen Class I Antigen-Processing Machinery Upregulation by Anticancer Therapies in the Era of Checkpoint Inhibitors: A Review. JAMA Oncol. 2022;8:462–73. 34940799 10.1001/jamaoncol.2021.5970 PMC8930447

[5] Zaretsky JM, Garcia-Diaz A, Shin DS, Escuin-Ordinas H, Hugo W, Hu-Lieskovan S, Torrejon DY, Abril-Rodriguez G, Sandoval S, Barthly L, et al. Mutations Associated with Acquired Resistance to PD-1 Blockade in Melanoma. N Engl J Med. 2016;375:819–29. 27433843 10.1056/NEJMoa1604958 PMC5007206

[6] Gettinger S, Choi J, Hastings K, Truini A, Datar I, Sowell R, Wurtz A, Dong W, Cai G, Melnick MA, et al. Impaired HLA Class I Antigen Processing and Presentation as a Mechanism of Acquired Resistance to Immune Checkpoint Inhibitors in Lung Cancer. Cancer Discov. 2017;7:1420–35. 29025772 10.1158/2159-8290.CD-17-0593 PMC5718941

[7] Sade-Feldman M, Jiao YJ, Chen JH, Rooney MS, Barzily-Rokni M, Eliane JP, Bjorgaard SL, Hammond MR, Vitzthum H, Blackmon SM, et al. Resistance to checkpoint blockade therapy through inactivation of antigen presentation. Nat Commun. 2017;8:1136. 29070816 10.1038/s41467-017-01062-w PMC5656607

[8] Beck S, Trowsdale J. The human major histocompatability complex: lessons from the DNA sequence. Annu Rev Genomics Hum Genet. 2000;1:117–37. 11701627 10.1146/annurev.genom.1.1.117

[9] Datar IJ, Hauc SC, Desai S, Gianino N, Henick B, Liu Y, Syrigos K, Rimm DL, Kavathas P, Ferrone S, Schalper KA. Spatial Analysis and Clinical Significance of HLA Class-I and Class-II Subunit Expression in Non-Small Cell Lung Cancer. Clin Cancer Res. 2021;27:2837–47. 33602682 10.1158/1078-0432.CCR-20-3655 PMC8734284

[10] Cerami E, Gao J, Dogrusoz U, Gross BE, Sumer SO, Aksoy BA, Jacobsen A, Byrne CJ, Heuer ML, Larsson E, et al. The cBio cancer genomics portal: an open platform for exploring multidimensional cancer genomics data. Cancer Discov. 2012;2:401–4. 22588877 10.1158/2159-8290.CD-12-0095 PMC3956037

[11] Gao J, Aksoy BA, Dogrusoz U, Dresdner G, Gross B, Sumer SO, Sun Y, Jacobsen A, Sinha R, Larsson E, et al: Integrative analysis of complex cancer genomics and clinical profiles using the cBioPortal. Sci Signal 2013, 6:pl1. 10.1126/scisignal.2004088 PMC4160307 23550210

[12] Wang X, Campoli M, Cho HS, Ogino T, Bandoh N, Shen J, Hur SY, Kageshita T, Ferrone S. A method to generate antigen-specific mAb capable of staining formalin-fixed, paraffin-embedded tissue sections. J Immunol Methods. 2005;299:139–51. 15896802 10.1016/j.jim.2005.02.006

[13] Schalper KA, Carvajal-Hausdorf D, McLaughlin J, Altan M, Velcheti V, Gaule P, Sanmamed MF, Chen L, Herbst RS, Rimm DL. Differential Expression and Significance of PD-L1, IDO-1, and B7–H4 in Human Lung Cancer. Clin Cancer Res. 2017;23:370–8. 27440266 10.1158/1078-0432.CCR-16-0150 PMC6350535

[14] Chang CC, Pirozzi G, Wen SH, Chung IH, Chiu BL, Errico S, Luongo M, Lombardi ML, Ferrone S. Multiple structural and epigenetic defects in the human leukocyte antigen class I antigen presentation pathway in a recurrent metastatic melanoma following immunotherapy. J Biol Chem. 2015;290:26562–75. 26381407 10.1074/jbc.M115.676130 PMC4646314

[15] Henick BS, Villarroel-Espindola F, Datar I, Sanmamed MF, Yu J, Desai S, Li A, Aguirre-Ducler A, Syrigos K, Rimm DL, et al. Quantitative tissue analysis and role of myeloid cells in non-small cell lung cancer. J Immunother Cancer. 2022;10:e005025. 35793873 10.1136/jitc-2022-005025 PMC9260844

[16] Krokhotin A, Du H, Hirabayashi K, Popov K, Kurokawa T, Wan X, Ferrone S, Dotti G, Dokholyan NV. Computationally Guided Design of Single-Chain Variable Fragment Improves Specificity of Chimeric Antigen Receptors. Mol Ther Oncolytics. 2019;15:30–7. 31650023 10.1016/j.omto.2019.08.008 PMC6804740

[17] Srivastava RM, Trivedi S, Concha-Benavente F, Hyun-Bae J, Wang L, Seethala RR. Branstetter BFt, Ferrone S, Ferris RL: STAT1-Induced HLA Class I Upregulation Enhances Immunogenicity and Clinical Response to Anti-EGFR mAb Cetuximab Therapy in HNC Patients. Cancer Immunol Res. 2015;3:936–45. 25972070 10.1158/2326-6066.CIR-15-0053 PMC4526378

[18] Langmead B, Salzberg SL. Fast gapped-read alignment with Bowtie 2. Nat Methods. 2012;9:357–9. 22388286 10.1038/nmeth.1923 PMC3322381

[19] Tarasov A, Vilella AJ, Cuppen E, Nijman IJ, Prins P. Sambamba: fast processing of NGS alignment formats. Bioinformatics. 2015;31:2032–4. 25697820 10.1093/bioinformatics/btv098 PMC4765878

[20] Li H, Handsaker B, Wysoker A, Fennell T, Ruan J, Homer N, Marth G, Abecasis G, Durbin R. Genome Project Data Processing S: The Sequence Alignment/Map format and SAMtools. Bioinformatics. 2009;25:2078–9. 19505943 10.1093/bioinformatics/btp352 PMC2723002

[21] Zhang Y, Liu T, Meyer CA, Eeckhoute J, Johnson DS, Bernstein BE, Nusbaum C, Myers RM, Brown M, Li W, Liu XS. Model-based analysis of ChIP-Seq (MACS). Genome Biol. 2008;9:R137. 18798982 10.1186/gb-2008-9-9-r137 PMC2592715

[22] Quinlan AR, Hall IM. BEDTools: a flexible suite of utilities for comparing genomic features. Bioinformatics. 2010;26:841–2. 20110278 10.1093/bioinformatics/btq033 PMC2832824

[23] Liao Y, Smyth GK, Shi W. featureCounts: an efficient general purpose program for assigning sequence reads to genomic features. Bioinformatics. 2014;30:923–30. 24227677 10.1093/bioinformatics/btt656

[24] Robinson MD, McCarthy DJ, Smyth GK. edgeR: a Bioconductor package for differential expression analysis of digital gene expression data. Bioinformatics. 2010;26:139–40. 19910308 10.1093/bioinformatics/btp616 PMC2796818

[25] Love MI, Huber W, Anders S. Moderated estimation of fold change and dispersion for RNA-seq data with DESeq2. Genome Biol. 2014;15:550. 25516281 10.1186/s13059-014-0550-8 PMC4302049

[26] Blum JS, Wearsch PA, Cresswell P. Pathways of antigen processing. Annu Rev Immunol. 2013;31:443–73. 23298205 10.1146/annurev-immunol-032712-095910 PMC4026165

[27] Schumacher TN, Heemels MT, Neefjes JJ, Kast WM, Melief CJ, Ploegh HL. Direct binding of peptide to empty MHC class I molecules on intact cells and in vitro. Cell. 1990;62:563–7. 2199065 10.1016/0092-8674(90)90020-f

[28] Song R, Porgador A, Harding CV. Peptide-receptive class I major histocompatibility complex molecules on TAP-deficient and wild-type cells and their roles in the processing of exogenous antigens. Immunology. 1999;97:316–24. 10447748 10.1046/j.1365-2567.1999.00759.x PMC2326830

[29] Brunnberg J, Barends M, Fruhschulz S, Winter C, Battin C, de Wet B, Cole DK, Steinberger P, Tampe R. Dual role of the peptide-loading complex as proofreader and limiter of MHC-I presentation. Proc Natl Acad Sci U S A. 2024;121:e2321600121. 38771881 10.1073/pnas.2321600121 PMC11145271

[30] Luft T, Rizkalla M, Tai TY, Chen Q, MacFarlan RI, Davis ID, Maraskovsky E, Cebon J. Exogenous peptides presented by transporter associated with antigen processing (TAP)-deficient and TAP-competent cells: intracellular loading and kinetics of presentation. J Immunol. 2001;167:2529–37. 11509592 10.4049/jimmunol.167.5.2529

[31] Tokunaga R, Zhang W, Naseem M, Puccini A, Berger MD, Soni S, McSkane M, Baba H, Lenz HJ. CXCL9, CXCL10, CXCL11/CXCR3 axis for immune activation - A target for novel cancer therapy. Cancer Treat Rev. 2018;63:40–7. 29207310 10.1016/j.ctrv.2017.11.007 PMC5801162

[32] Waldmann TA, Miljkovic MD, Conlon KC: Interleukin-15 (dys)regulation of lymphoid homeostasis: Implications for therapy of autoimmunity and cancer. J Exp Med 2020, 217. 10.1084/jem.20191062 PMC7037239 31821442

[33] Liau NPD, Laktyushin A, Lucet IS, Murphy JM, Yao S, Whitlock E, Callaghan K, Nicola NA, Kershaw NJ, Babon JJ. The molecular basis of JAK/STAT inhibition by SOCS1. Nat Commun. 2018;9:1558. 29674694 10.1038/s41467-018-04013-1 PMC5908791

[34] He Y, Zhang W, Zhang R, Zhang H, Min W. SOCS1 inhibits tumor necrosis factor-induced activation of ASK1-JNK inflammatory signaling by mediating ASK1 degradation. J Biol Chem. 2006;281:5559–66. 16407264 10.1074/jbc.M512338200

[35] Ho J, Pelzel C, Begitt A, Mee M, Elsheikha HM, Scott DJ, Vinkemeier U. STAT2 Is a Pervasive Cytokine Regulator due to Its Inhibition of STAT1 in Multiple Signaling Pathways. PLoS Biol. 2016;14:e2000117. 27780205 10.1371/journal.pbio.2000117 PMC5079630

[36] Canar J, Darling K, Dadey R, Gamero AM. The duality of STAT2 mediated type I interferon signaling in the tumor microenvironment and chemoresistance. Cytokine. 2023;161:156081. 36327541 10.1016/j.cyto.2022.156081 PMC9720715

[37] Cai L, Michelakos T, Yamada T, Fan S, Wang X, Schwab JH, Ferrone CR, Ferrone S. Defective HLA class I antigen processing machinery in cancer. Cancer Immunol Immunother. 2018;67:999–1009. 29487978 10.1007/s00262-018-2131-2 PMC8697037

[38] Pereira C, Gimenez-Xavier P, Pros E, Pajares MJ, Moro M, Gomez A, Navarro A, Condom E, Moran S, Gomez-Lopez G, et al. Genomic Profiling of Patient-Derived Xenografts for Lung Cancer Identifies B2M Inactivation Impairing Immunorecognition. Clin Cancer Res. 2017;23:3203–13. 28302866 10.1158/1078-0432.CCR-16-1946

[39] Keusekotten K, Leonhardt RM, Ehses S, Knittler MR. Biogenesis of functional antigenic peptide transporter TAP requires assembly of pre-existing TAP1 with newly synthesized TAP2. J Biol Chem. 2006;281:17545–51. 16624807 10.1074/jbc.M602360200

[40] Antoniou AN, Ford S, Pilley ES, Blake N, Powis SJ. Interactions formed by individually expressed TAP1 and TAP2 polypeptide subunits. Immunology. 2002;106:182–9. 12047747 10.1046/j.1365-2567.2002.01415.x PMC1782706

[41] Padariya M, Kote S, Mayordomo M, Dapic I, Alfaro J, Hupp T, Fahraeus R, Kalathiya U. Structural determinants of peptide-dependent TAP1-TAP2 transit passage targeted by viral proteins and altered by cancer-associated mutations. Comput Struct Biotechnol J. 2021;19:5072–91. 34589184 10.1016/j.csbj.2021.09.006 PMC8453138

[42] Abele R, Tampe R. Moving the Cellular Peptidome by Transporters. Front Cell Dev Biol. 2018;6:43. 29761100 10.3389/fcell.2018.00043 PMC5937356

[43] Alspach E, Lussier DM, Schreiber RD. Interferon gamma and Its Important Roles in Promoting and Inhibiting Spontaneous and Therapeutic Cancer Immunity. Cold Spring Harb Perspect Biol. 2019;11:a028480. 29661791 10.1101/cshperspect.a028480 PMC6396335

[44] Li Z, Jiang J, Wang Z, Zhang J, Xiao M, Wang C, Lu Y, Qin Z. Endogenous interleukin-4 promotes tumor development by increasing tumor cell resistance to apoptosis. Cancer Res. 2008;68:8687–94. 18974110 10.1158/0008-5472.CAN-08-0449

[45] Todaro M, Lombardo Y, Francipane MG, Alea MP, Cammareri P, Iovino F, Di Stefano AB, Di Bernardo C, Agrusa A, Condorelli G, et al. Apoptosis resistance in epithelial tumors is mediated by tumor-cell-derived interleukin-4. Cell Death Differ. 2008;15:762–72. 18202702 10.1038/sj.cdd.4402305

[46] Rakaee M, Busund LR, Jamaly S, Paulsen EE, Richardsen E, Andersen S, Al-Saad S, Bremnes RM, Donnem T, Kilvaer TK. Prognostic Value of Macrophage Phenotypes in Resectable Non-Small Cell Lung Cancer Assessed by Multiplex Immunohistochemistry. Neoplasia. 2019;21:282–93. 30743162 10.1016/j.neo.2019.01.005 PMC6369140

[47] Mandruzzato S, Solito S, Falisi E, Francescato S, Chiarion-Sileni V, Mocellin S, Zanon A, Rossi CR, Nitti D, Bronte V, Zanovello P. IL4Ralpha+ myeloid-derived suppressor cell expansion in cancer patients. J Immunol. 2009;182:6562–8. 19414811 10.4049/jimmunol.0803831

[48] Fu C, Jiang L, Hao S, Liu Z, Ding S, Zhang W, Yang X, Li S. Activation of the IL-4/STAT6 Signaling Pathway Promotes Lung Cancer Progression by Increasing M2 Myeloid Cells. Front Immunol. 2019;10:2638. 31798581 10.3389/fimmu.2019.02638 PMC6863933

[49] Kawakami M, Kawakami K, Stepensky VA, Maki RA, Robin H, Muller W, Husain SR, Puri RK. Interleukin 4 receptor on human lung cancer: a molecular target for cytotoxin therapy. Clin Cancer Res. 2002;8:3503–11. 12429641

[50] Maier B, Leader AM, Chen ST, Tung N, Chang C, LeBerichel J, Chudnovskiy A, Maskey S, Walker L, Finnigan JP, et al. A conserved dendritic-cell regulatory program limits antitumour immunity. Nature. 2020;580:257–62. 32269339 10.1038/s41586-020-2134-y PMC7787191

[51] LaMarche NM, Hegde S, Park MD, Maier BB, Troncoso L, Le Berichel J, Hamon P, Belabed M, Mattiuz R, Hennequin C, et al. An IL-4 signalling axis in bone marrow drives pro-tumorigenic myelopoiesis. Nature. 2024;625:166–74. 38057662 10.1038/s41586-023-06797-9 PMC11189607

[52] Galluzzi L, Humeau J, Buque A, Zitvogel L, Kroemer G. Immunostimulation with chemotherapy in the era of immune checkpoint inhibitors. Nat Rev Clin Oncol. 2020;17:725–41. 32760014 10.1038/s41571-020-0413-z

[53] Allam M, Hu T, Lee J, Aldrich J, Badve SS, Gokmen-Polar Y, Bhave M, Ramalingam SS, Schneider F, Coskun AF. Spatially variant immune infiltration scoring in human cancer tissues. NPJ Precis Oncol. 2022;6:60. 36050391 10.1038/s41698-022-00305-4 PMC9437065

