# Supplementary material for: IL-4 mediated TAP2 downregulation is a dominant and reversible mechanism of immune evasion and immunotherapy resistance in non-small cell lung cancer
Source: Mol Cancer. 2025 Mar 17;24:80. doi: 10.1186/s12943-025-02276-z (PMC11912681; doi:10.1186/s12943-025-02276-z)
Supplement: Supplementary file 1 — Supplementary Material 1. [file 12943_2025_2276_MOESM1_ESM.doc]

**Supplementary Materials**

**IL-4 mediated TAP2 downregulation is a dominant and reversible mechanism of immune evasion and immunotherapy resistance in non-small cell lung cancer**

Kishu Ranjan^1^, Barani Kumar Rajendran^1^, Imad Ud Deen^1^, Adrien Costantini^1^, Miguel Lopez de Rodas^1^, Shruti S. Desai^1^, Frankie Scallo^1^, Nicole Gianino^1^, Soldano Ferrone^2^, Kurt A. Schalper^1*^

^1^Department of Pathology, School of Medicine, Yale University, New Haven, 06520, CT, USA

^2^Department of Surgery, Massachusetts General Hospital and Harvard Medical School, Boston, 02114, MA, USA

**Correspondence to**:

Kurt A. Schalper, MD, PhD

Associate Professor

Brady Memorial Laboratory,

310 Cedar Street, Ste BML, Rm 113

New Haven, CT 06510

Phone: 203-737-4205

Email: kurt.schalper@yale.edu

| **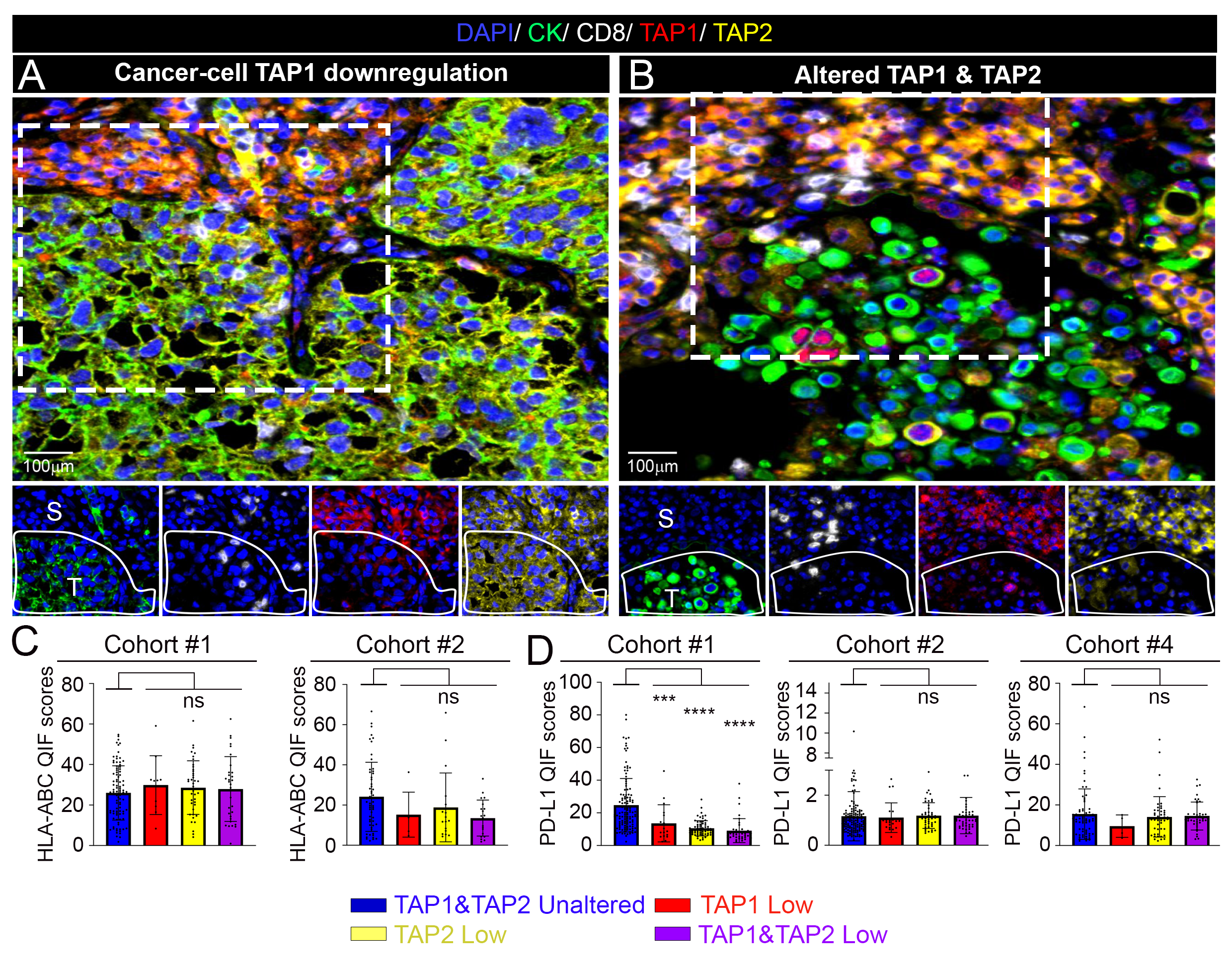Fig. S1:** **Cancer cell selective downregulation of TAP1 and TAP1/2 in NSCLC.** (**A-B**) Representative multicolor immunofluorescence microphotographs showing the simultaneous detection of cytokeratin (CK) positive tumor epithelial cells (green), CD8^+^ TILs (white), TAP1 (red) and TAP2 (yellow) proteins in human NSCLC. Cell nuclei were stained with DAPI (blue). (**C)** Cancer cell expression of HLA-ABC stratified across cohorts 1 and 2. (**D)**, Cancer cell expression of PD-L1 stratified across cohorts #1, #2 and #4. QIF scores are shown as thousands of fluorescence units. Data presented as the mean ± s.d for C-D, ***, p<0.001; ****, p<0.0001 and determined by two-tailed unpaired Student’s t-test. ns, not significant; QIF, quantitative immunofluorescence; S, stroma; T, tumor. |
| --- |

| **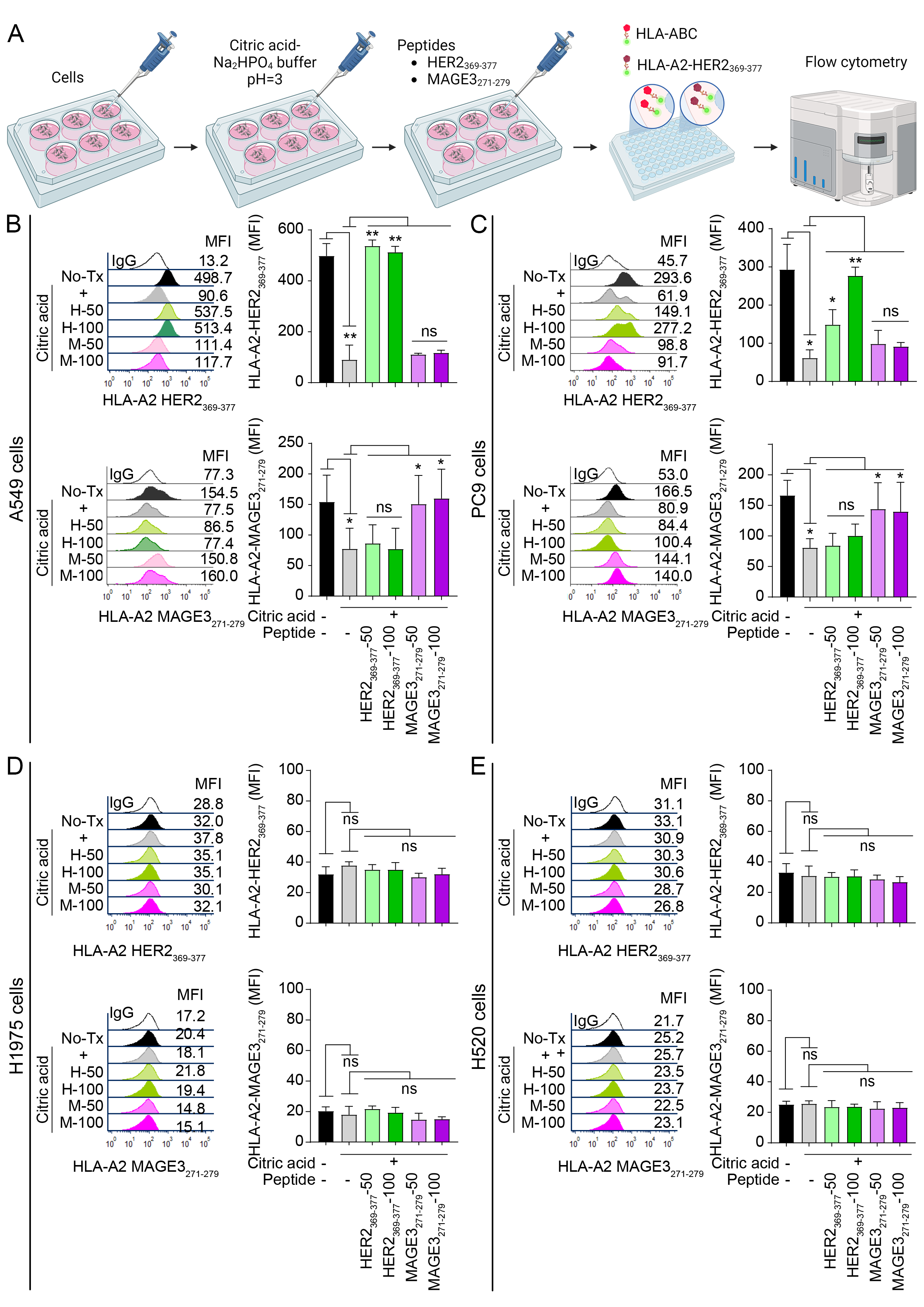** |
| --- |
| **Fig. S2:** **Acid stripping and reloading of peptides to cells.** (**A**) Outline of the experimental strategy used to eliminate peptide bounds to surface HLA-I complexes using citric acid solution and measure changes in the levels of selected HLA-peptide complexes in lung cancer cells by flow cytometry. (**B-E**) Cells were acid-stripped followed by incubation with HER2_369-371_ peptides (50µM or 100 µM) or MAGE3_271-279_ peptides (50µM or 100 µM) and analyzed for surface HLA-A2-HER2_369-377_ and HLA-A2-MAGE3_271-279_ complex levels by flow cytometry. An isotype control antibody (IgG) was used as a background signal reference. Data presented as the mean ± s.d.; *, p<0.05; **, p<0.01 determined by two-tailed unpaired Student’s t-test with a Holm-Bonferroni correction for multiple comparisons. MFI, mean fluorescent intensity; ns, not significant; Tx, treatment. |
| 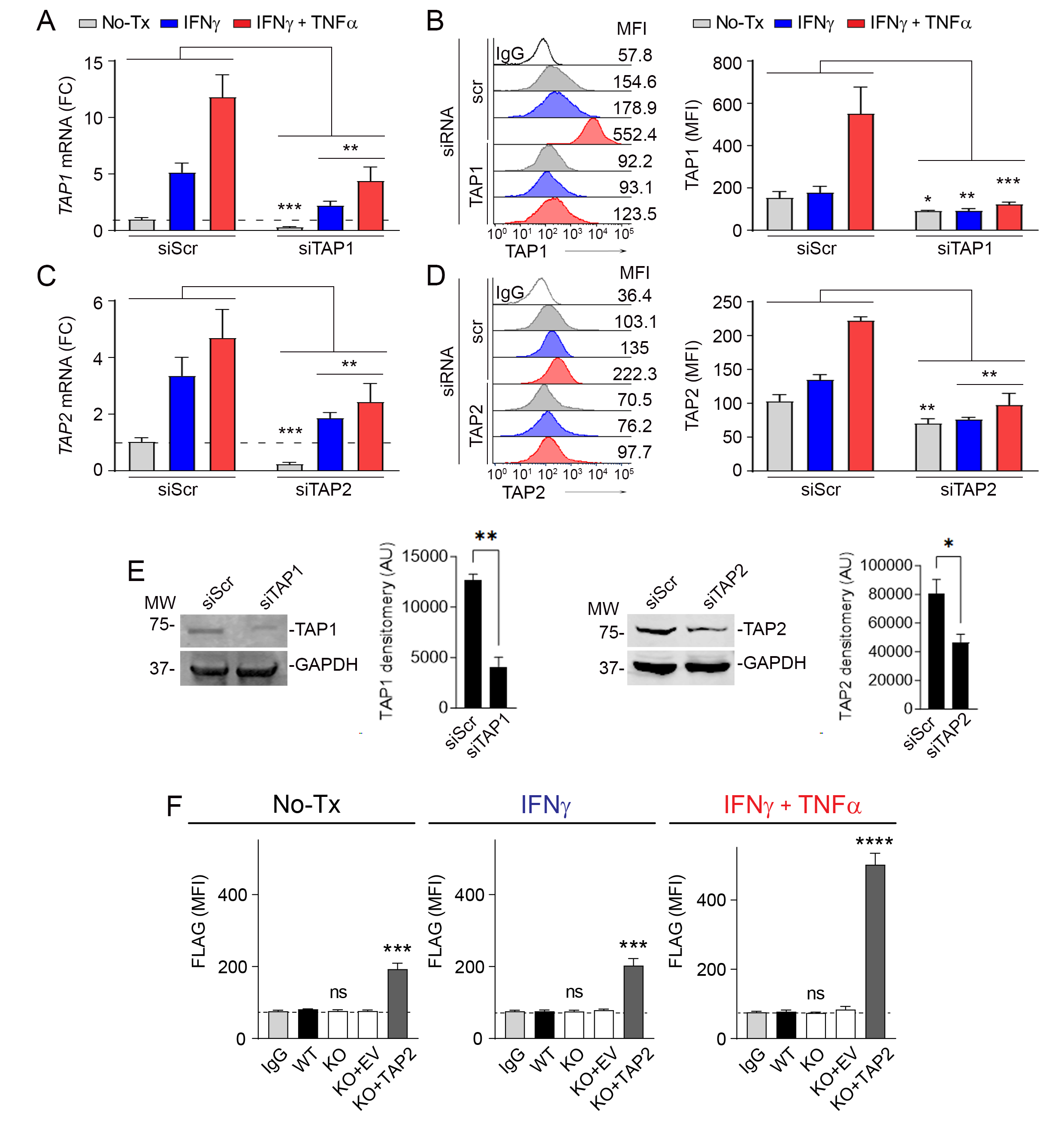**Fig. S3:** ***TAP1* and *TAP2* knockdown validation. (A-E)** A549 lung cancer cells were transfected with scrambled/control siRNA or with *TAP1* and/or *TAP2* targeting siRNAs and left untreated or stimulated with IFNγ or IFNγ + TNFα. B and C, mRNA expression after 8 hours by qRT-PCR. B and D, TAP1 and TAP2 protein expression by flow cytometry. E, TAP1 and TAP2 protein expression by Western blot analysis (**F)** A549 *TAP2* knockout (KO) cells were transfected with an empty vector (KO+EV) or with a vector containing FLAG-TAP2 (KO+TAP2) and left untreated (black) or stimulated with cytokines IFNγ (blue) or IFNγ plus TNFα (red). TAP2 protein expression by staining the FLAG tag and analyzed by flow cytometry. For panels B, D and F, an isotype control antibody (IgG) was used as a background signal reference. Data presented as the mean ± s.d.; *, p<0.05; **, p<0.01; ***, p<0.001 determined by two-tailed unpaired Student’s t-test with a Holm-Bonferroni correction for multiple comparisons. For panels B and D, Scr transfected cells were used as a control for statistical comparison, and for F parental wild type (WT) cells were compared with *TAP2* deleted cells (KO) and *TAP2* deleted plus EV (KO+EV) were compared with *TAP2* deleted with posterior *TAP2* transfection (KO+TAP2). MFI, mean fluorescent intensity; si, siRNA; scr, scrambled; Tx, treatment; ns, not significant.   | **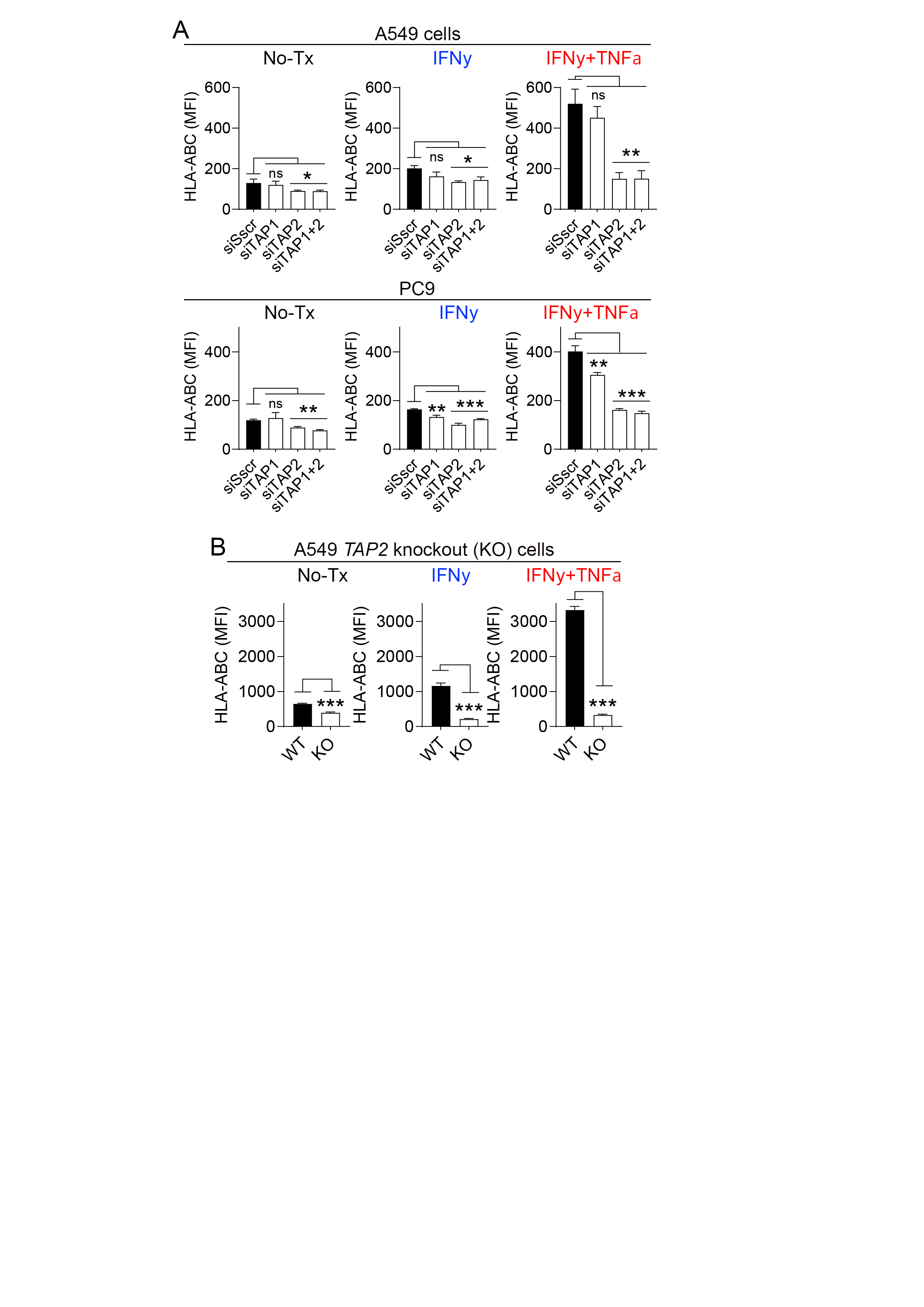Fig. S4:** **Loss of TAP2 reduces surface HLA-ABC levels. (A**) A549 or PC9 cells were transfected with scrambled siRNA (siScr) or *TAP1* siRNA (siTAP1) or *TAP2* siRNA (siTAP2) and incubated for 48 h and, post transfection cells were treated with IFNγ or INFγ + TNFα and analyzed for the surface expression of HLA-ABC by flow cytometry. (**B)** A549 wild type (WT) or *TAP2* knockout (KO) cells were treated with IFNγ or INFγ + TNFα and analyzed for the surface expression of HLA-ABC by flow cytometry. An isotype control antibody (IgG) was used as a background signal reference. Data is presented as the mean ± s.d.; *, p<0.05; **, p<0.01; ***, p<0.001 determined by two-tailed unpaired Student’s t-test. MFI, mean fluorescent intensity; ns, not significant; Tx, treatment. | | --- |  | **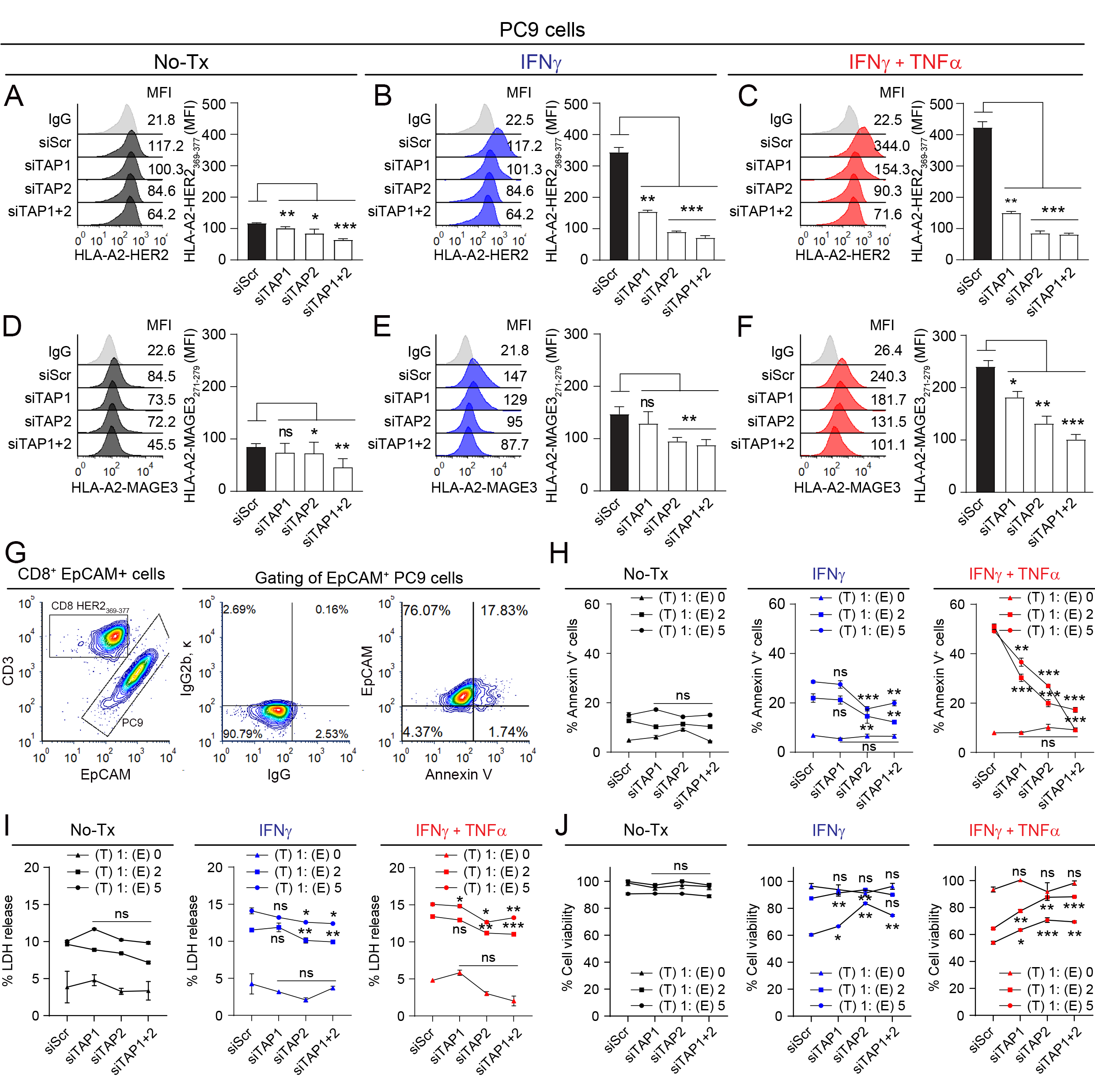** | | --- | | **Fig. S5: TAP1 and TAP2 downregulation and tumor cell immunogenicity in PC9 cells. (A-F**) PC9 lung cancer cells were transfected with scrambled/control siRNA or with *TAP1* and/or *TAP2* targeting siRNAs and left untreated (black histograms) or stimulated with IFNγ (blue histograms) or IFNγ + TNFα (red histograms). Panels A-C show the surface levels of HLA-A2-HER2_369-377_, and panels D-F show the levels of HLA-A2-MAGE3_271-279_. (**G-J**) PC9 lung cancer cells were transfected with scrambled siRNA or with *TAP1* and/or *TAP2* targeting siRNAs; and left untreated (black) or stimulated with IFNγ (blue) or IFNγ + TNFα (red). Post treatment, target lung tumor cells were co-cultured with effector (CD8^+^ T-cells) cells in the ratios of 1:0, 1:2 and 1:5, respectively. Panel G represents flow cytometry gating strategy to assess cell apoptosis in cancer cell/T-cell co-cultures using the markers CD3 (for CD8 T-cells), EpCAM (for tumor cells) and Annexin V. Panel H show the percentage of EpCAM^+^ and Annexin V^+^ apoptotic cancer cells. Panels I show the percent of LDH release, and panels J represent the cellular viability using MTT assay. An isotype control antibody (IgG) was used as a background signal reference. Data is presented as the mean ± s.d.; *, p<0.05; **, p<0.01; ***, p<0.001 determined by two-tailed unpaired Student’s t-test with a Holm-Bonferroni correction for multiple comparisons. MFI, mean fluorescent intensity; ns, not significant; Tx, treatment. |  | **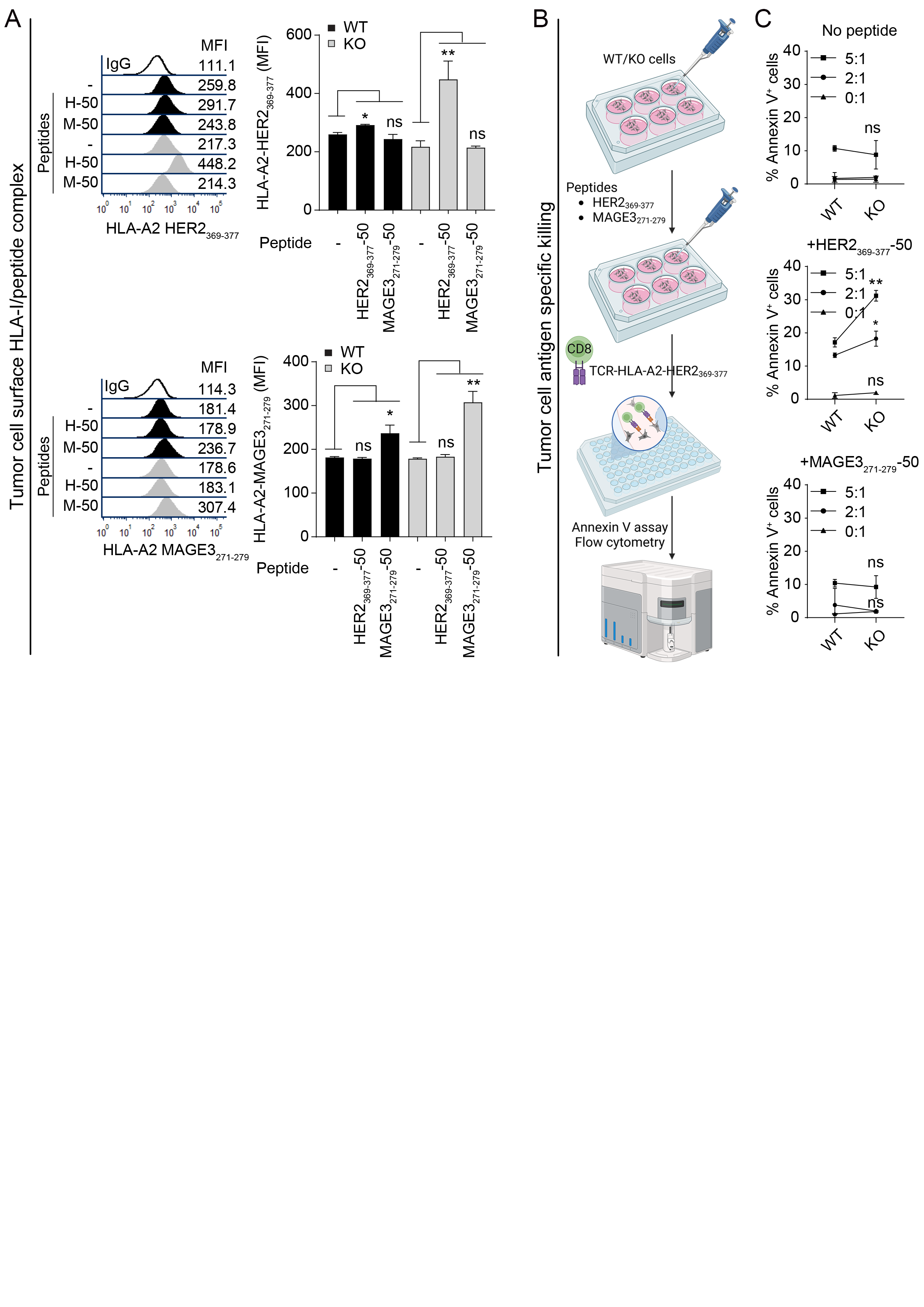** | | --- | | **Fig. S6: Exogenous antigenic peptides stabilize surface HLA-class I/peptide complexes. (A)** A549 wild type (WT) or *TAP2* knockout (KO) cells were loaded with 50µM of HER2_369-377_ nonamer or MAGE3_271-277_ nonamer peptides and surface expression of HLA-A2/peptide complexes was analyzed by flow cytometry. (**B**) schematic of A549 (WT/KO) cells with CD8^+^ T-cells in a co-culture and (**C**) A549 lung tumor target (T) cells were co-cultured with effector (CD8^+^ T-cell) cell in the ratios of 1:0, 1:2 and 1:5, respectively. Annexin V assay was used to measure percent cell death. For A and C, an isotype control antibody (IgG) was used as a background signal reference. Data is presented as the mean ± s.d.; *, p<0.05; **, p<0.01 determined by two-tailed unpaired Student’s t-test with a Holm-Bonferroni correction for multiple comparisons. MFI, mean fluorescent intensity; ns, not significant; Tx, treatment. | |

| 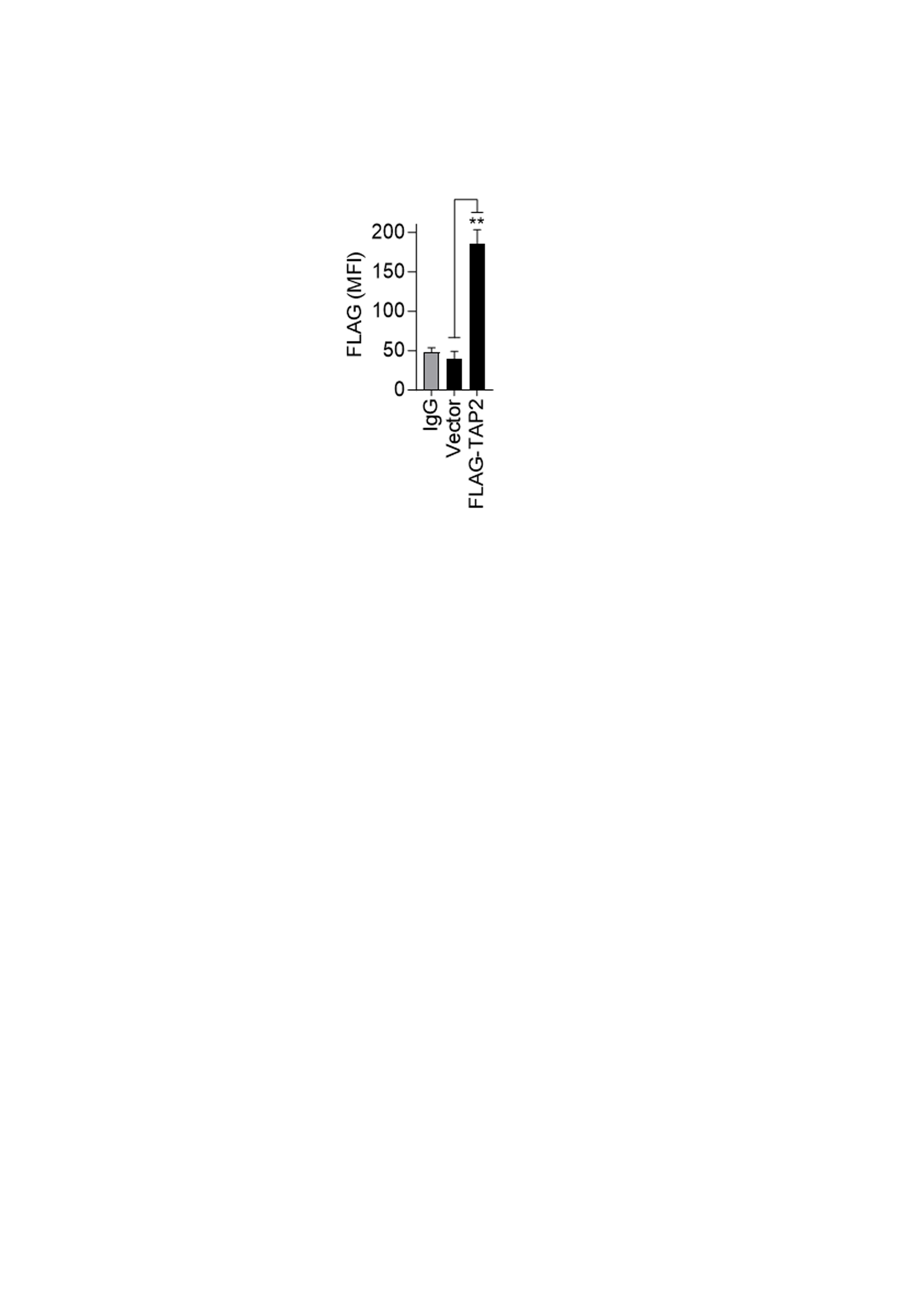 |
| --- |
| **Fig. S7: Transient expression of *TAP2* in A549 cells.** Cells were transfected with an empty vector or with a vector containing FLAG-*TAP2* (FLAG-TAP2) and stained with FLAG antibody to measure TAP2 expression by flow cytometry. An isotype control antibody (IgG) was used as a background signal reference. Data presented as the mean ± s.d.; **, p<0.01 determined by two-tailed unpaired Student’s t-test. MFI, mean fluorescent intensity. |

| **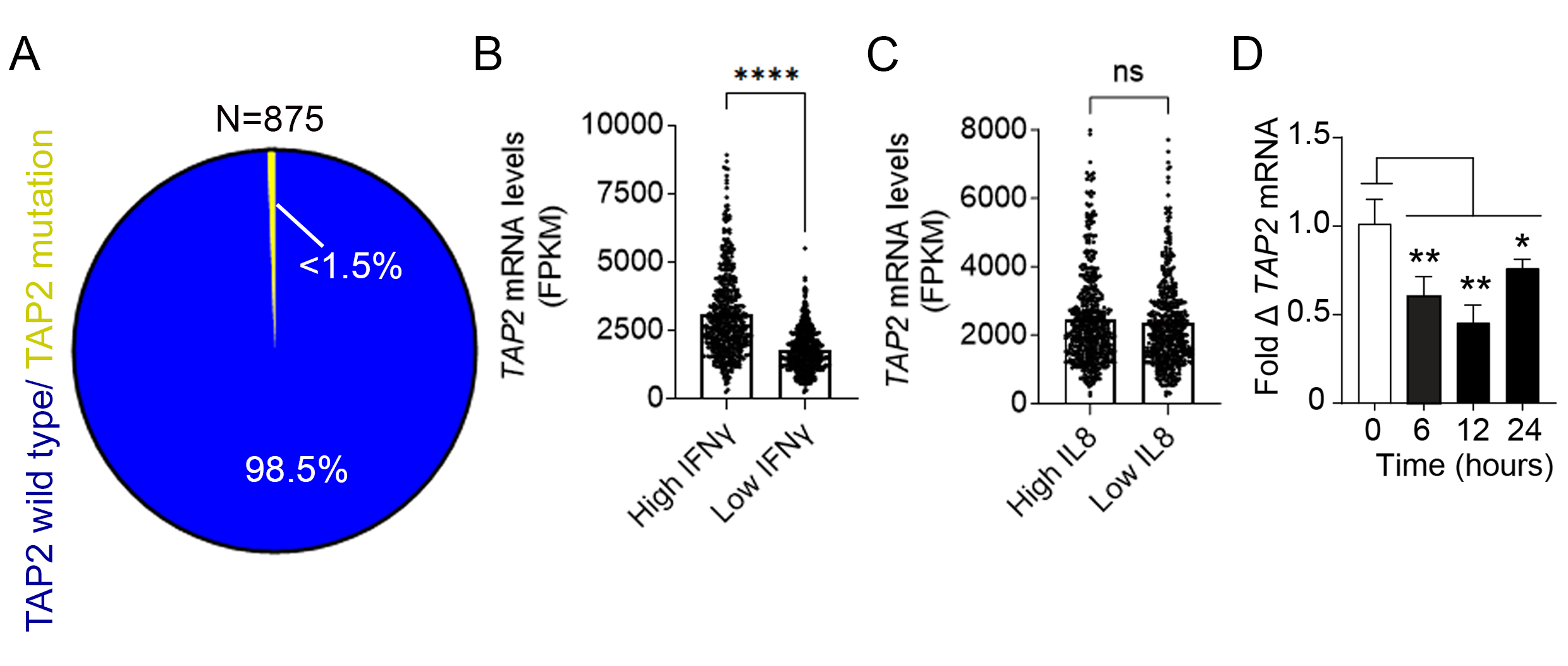** |
| --- |
| **Fig. S8:** **Genomic/transcriptomic analysis of** **NSCLCs from TCGA**. **(A)** Frequency of deleterious *TAP2* gene mutations (including missense, indels, deep deletion) in NSCLC samples from the TCGA collection obtained from cBioPortal. The frequency of cases with wild type and mutated *TAP2* are indicated with white-colored text within the pie chart. **(B-C)** *TAP2* mRNA expression stratified with the median cut-off of B, *IFNγ* and C*, IL-8* mRNA expressions in NSCLC cases from TCGA cohort. (D) A549 cells were treated with IL-4 for 0-24h and *TAP2* mRNA fold changes were measured using qRT-PCR. Data presented as the mean ± s.d.; *, p<0.05; **, p<0.01; ****, p<0.0001 determined by two-tailed unpaired Student’s t-test. FPKM, fragments per kilobase million; ns, not significant. |
| **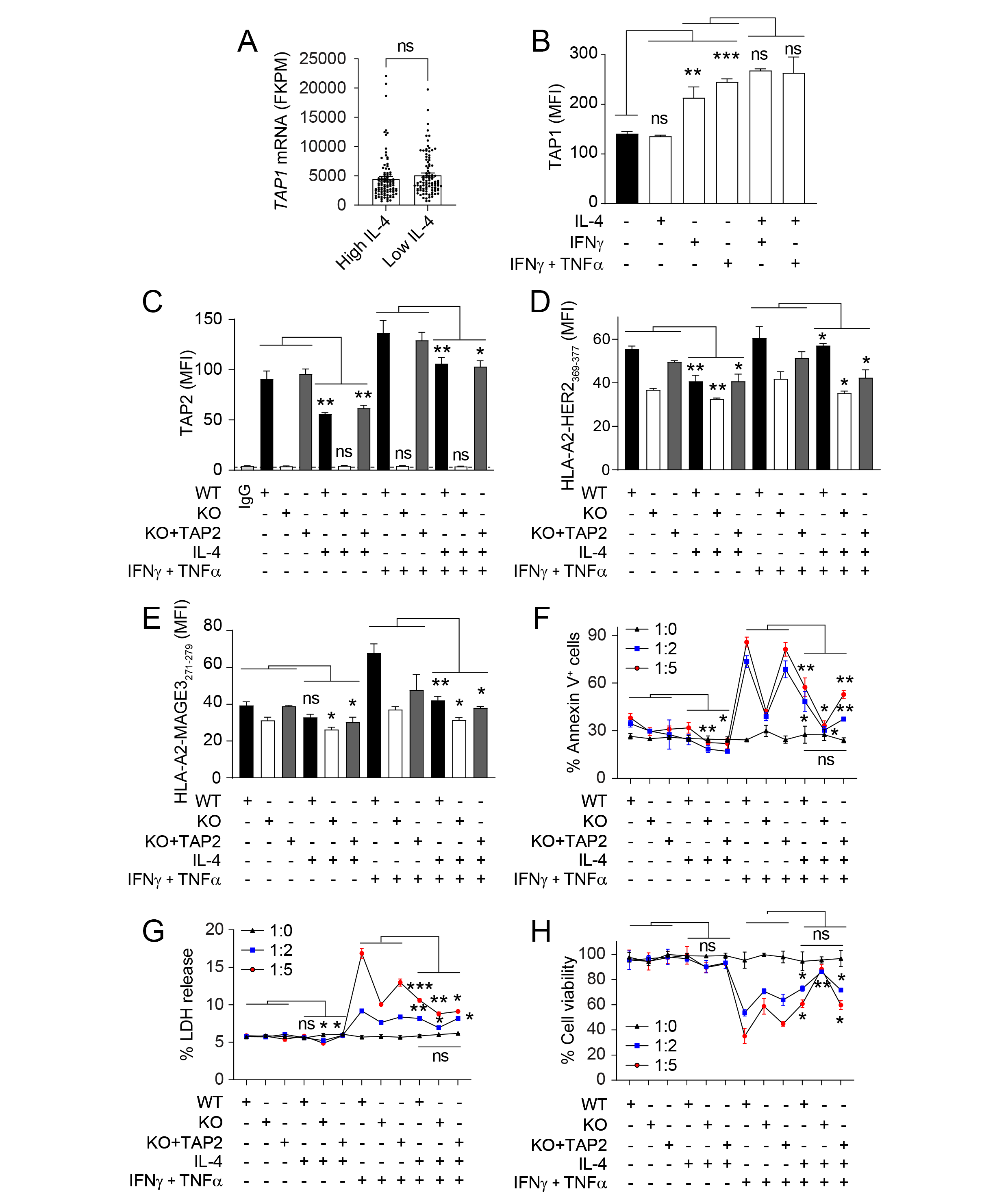Fig. S9:** **IL-4 treatment modulates surface tumor antigenicity and T-cell killing. (A)** TCGA NSCLC cohort analysis of *TAP1* expression levels stratified by the median *IL-4* mRNA. (**B**) A549 cells were treated with IL-4, IL-4 + IFNγ or IL-4 with IFNγ + TNFα, and TAP1 expression was measured using flow cytometry. (**C-H**) A549 *TAP2* knockout (KO) cells transfected with empty vector (KO+EV) or FLAG-*TAP2* (KO+TAP2) and left untreated or treated with IL-4 or IL-4 with IFNγ + TNFα, C, endogenous TAP2 expression by flow, D, surface HLA-A2-HER2_369-377_ levels and E, surface levels of HLA-A2-MAGE3_271-279_.  F, post cytokine treatment, cells were washed and co-cultured with tumor antigen-specific CD8 T-cells at different target cell (tumor): effector (CD8^+^ T-cell) cell ratios and studied using Annexin V staining, G, % LDH release and H, MTT cell viability assay. For panel B-H, an isotype control antibody (IgG) was used as a background signal reference. Data presented as the mean ± s.d.; *, p<0.05; **, p<0.01; ***, p<0.001 determined by two-tailed unpaired Student’s t-test with a Holm-Bonferroni for multiple comparison. ns, not significant; h, hours; MFI, mean fluorescent intensity. |

| **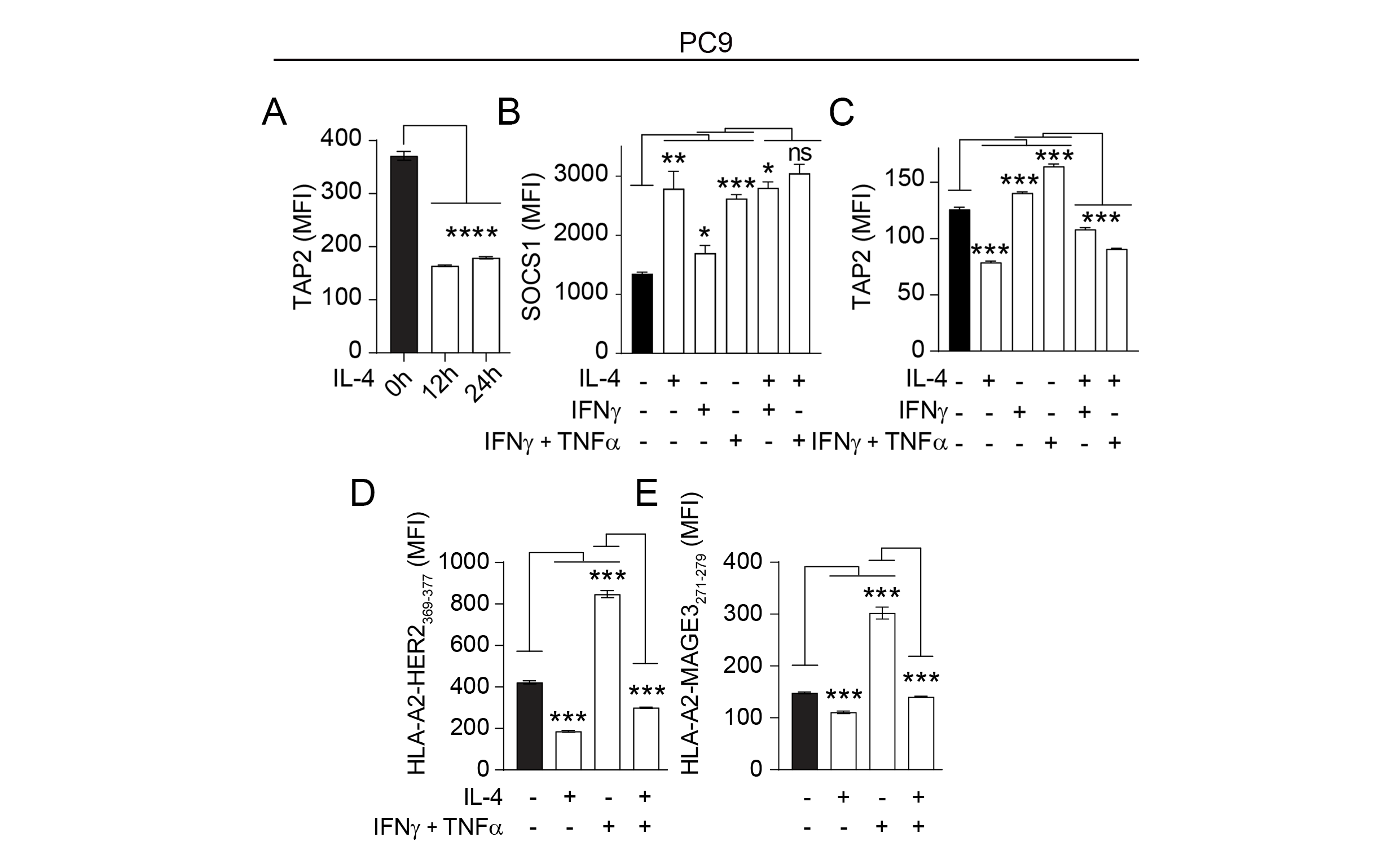** |
| --- |
| **Fig. S10:** **IL-4 modulates the expression of TAP2 in PC9 cells**. (**A**) PC9 cells treated with IL-4 for 0-24 hours and TAP2 expression analyzed by flow cytometry. (**B-E**) PC9 cells were treated with IL-4 or IL-4 + IFNγ or IL-4 with IFNγ + TNFα, B, TAP2 expression and C, SOCS1 expression, D, surface HLA-A2-HER2_369-377_ and E, surface HLA-A2-MAGE3_271-279_ expression were analyzed by flow cytometry. An isotype control antibody (IgG) was used as a background signal reference. Data presented as the mean ± s.d.; *, p<0.05; **, p<0.01; ***, p<0.001; ****, p<0.0001 determined by two-tailed unpaired Student’s t test with a Holm-Bonferroni correction for multiple comparisons. ns, not significant; h, hours; MFI, mean fluorescent intensity. |
|  |
|  |

| **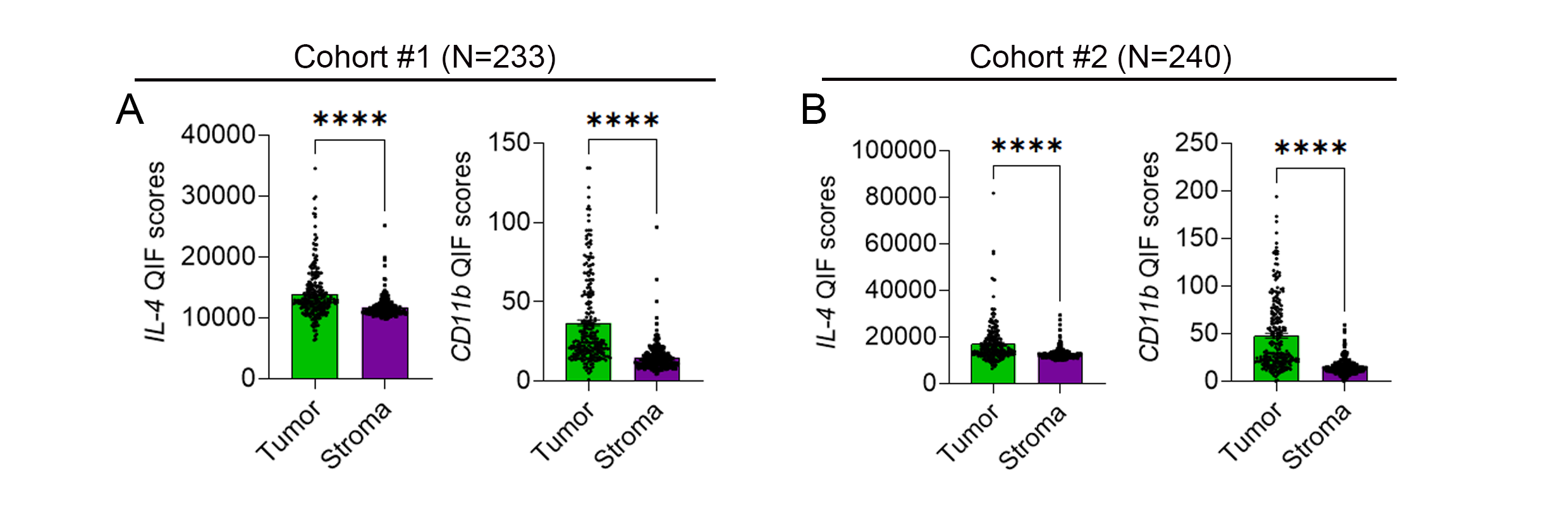** |
| --- |
| **Fig. S11:** **IL-4 protein levels in cancer cell nests.** **(A-B)** Spatially resolved expression levels of *IL-4* mRNA measured selectively in CK^+^ tumor cells or CK-negative (non-malignant) stromal cells in 2 retrospective NSCLC cohorts related to Figure 5L-M. Data are presented as the mean ± s.d. ****, p<0.0001 determined by two-tailed unpaired Student’s t-test. |

| **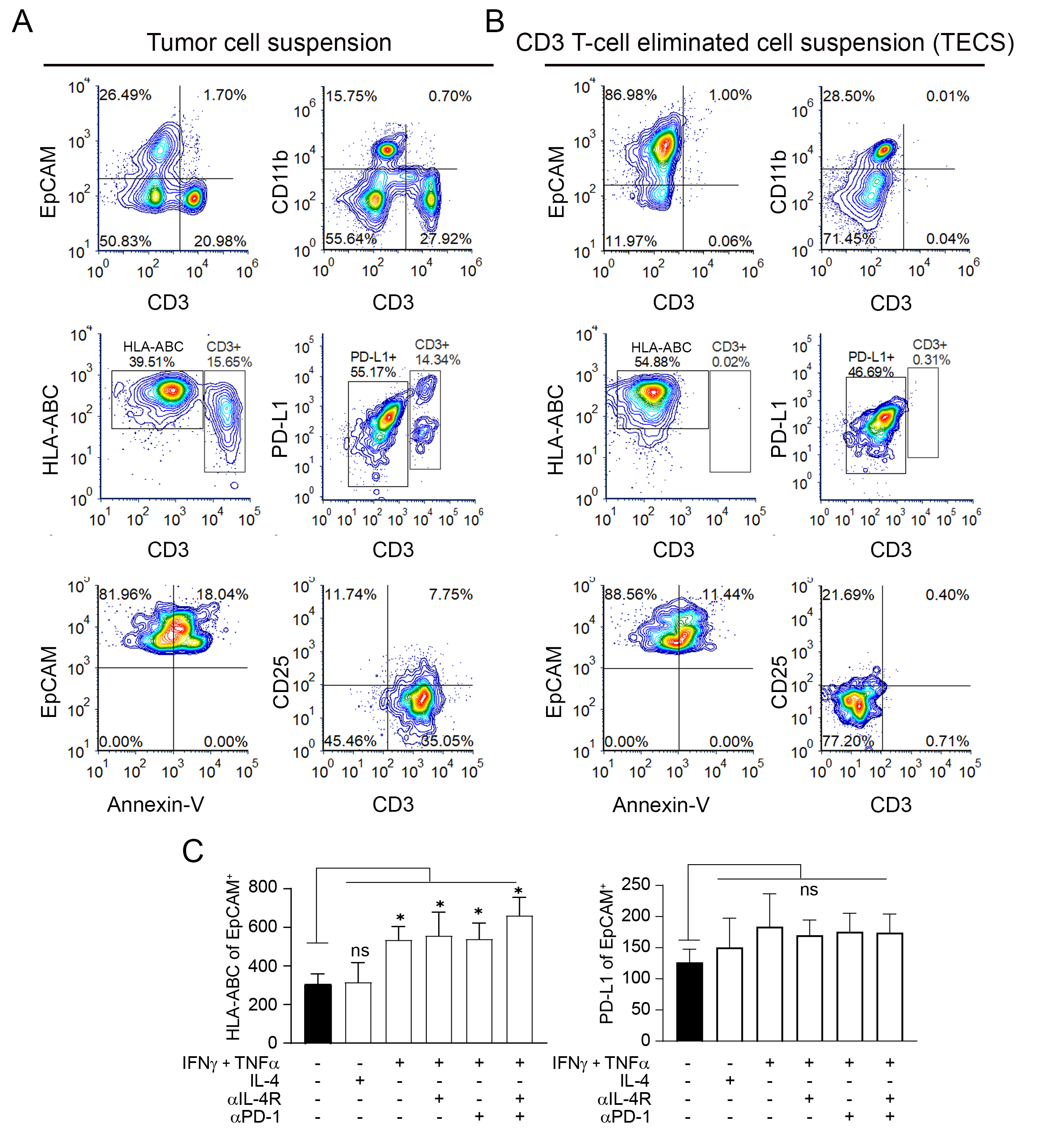Fig. S12:** **Preparation of T-cells eliminated single cell suspension (TECS) from human NSCLC tissues.** (**A-B**) Contour plots showing the levels of multiple markers in NSCLC single cell suspensions before and after T-cell elimination and analyzed by flow cytometry. (**C**) Autologous single cell suspension cultures including cancer and immune cells from primary NSCLC tissues stimulated with IFNγ + TNFα followed by incubation with αIL-4R or Pembrolizumab (αPD-1). The levels of EpCAM^+^/HLA-ABC^+^ cells and EpCAM^+^/PD-L1^+^ cells were measured by flow cytometry. Isotype (IgG) was used as a background control. Data is presented as the mean ± s.d.; *, p<0.05. ns, not significant; MFI, mean fluorescent intensity. |
| --- |
| | 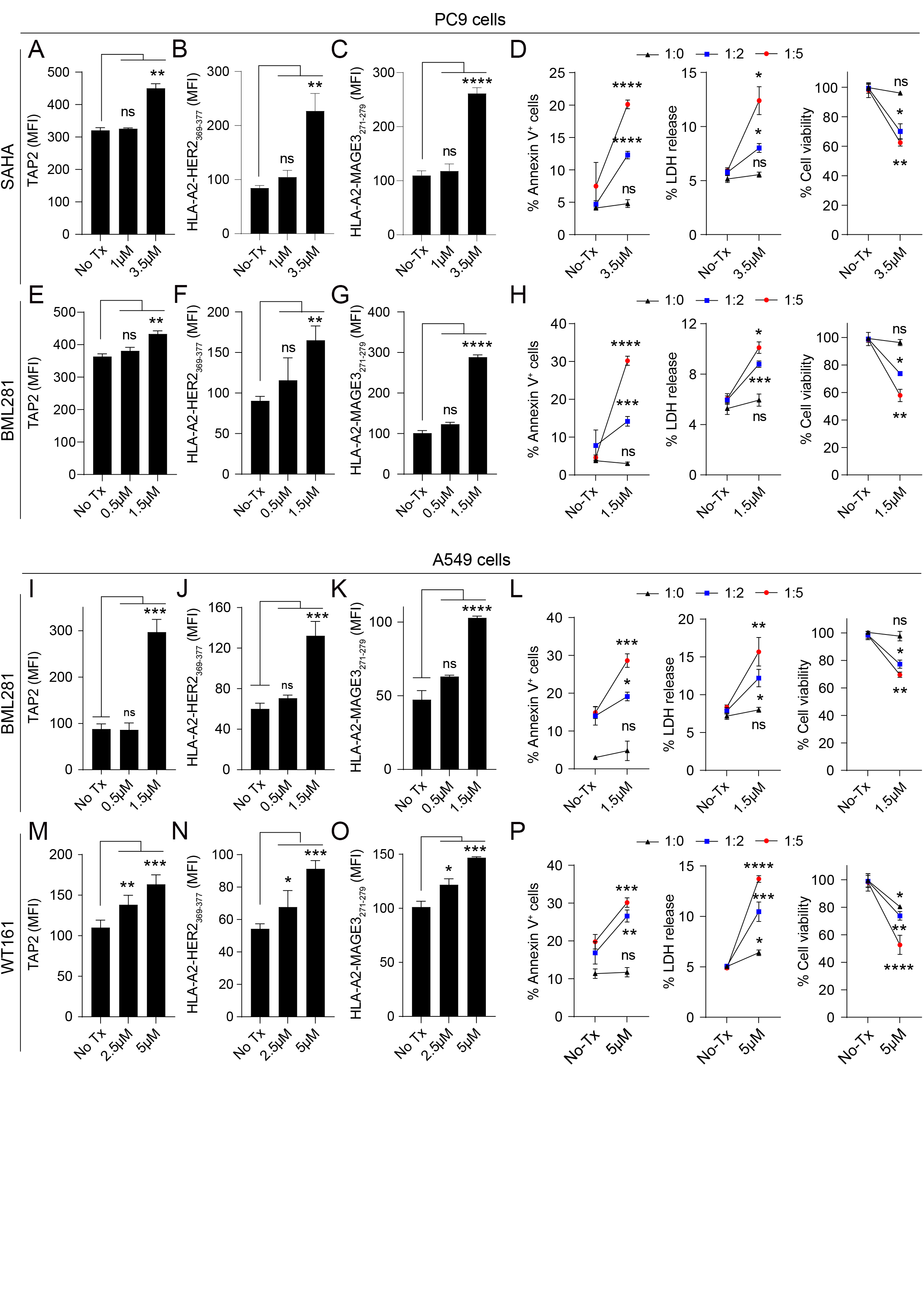**Fig. S13:** **Restoration of TAP2 protein expression and tumor cell surface antigenicity.** (**A-P**) A549 or PC9 cells were treated with SAHA or BML281 or WT161. (A, E, I, M) endogenous TAP2 expression, (B, F, J, N) surface levels of HLA-A2-HER2_369-377_, (C, G, K, O) surface levels of HLA-A2-MAGE3_271-279_, and (D, H, L, P) levels of apoptotic cell death (Annexin V staining) and cell viability (LDH release and MTT assay) of parental A549/PC9 cells co-incubated with tumor antigen-specific effector CD8^+^ T-cells using different effector to target cell ratios (1:0, 1:2 and 1:5) with or without treatment with different concentrations of the indicated compounds. An isotype control antibody (IgG) was used as a background reference. Data is presented as the mean ± s.d.; *p<0.05; **p<0.01; ***, p<0.001; ****, p<0.0001 determined by two-tailed unpaired Student’s t-test. ns, non-significant; No-Tx, no treatment; MFI, mean fluorescent intensity.  .   | **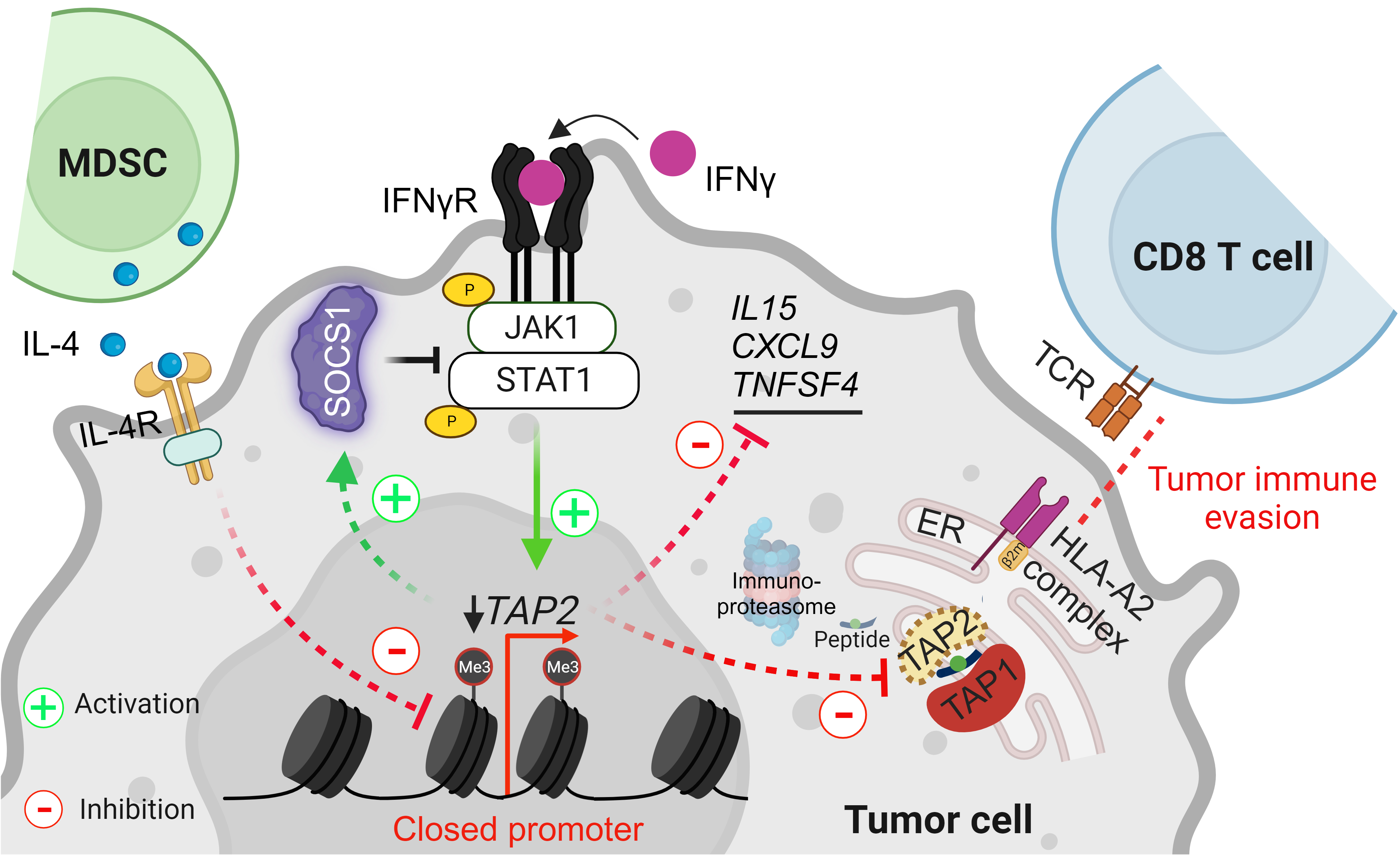** | | --- | | **Fig. S14:** **Graphical model summarizing the IL-4 mediated epigenetic regulation of TAP2 in lung cancer.** IL-4 signaling through IL-4R epigenetically suppresses *TAP2* expression leading to cancer cell adaptive immune evasion. Downregulation of TAP2 induces SOCS1 expression, suppresses IFN gamma pathway activation, limits sensitivity to proinflammatory cytokines and reduces the levels of antigenic HLA-peptide complexes in malignant cells. | | | --- | --- | --- | |  | |  | |
|  |

**Table S1:** Real time qPCR primer details.

| **Genes** | **Primers** |
| --- | --- |
| Human *TAP1* | Forward: 5’- TACAAGATGGCTCAGCCGATA -3’  Reverse: 5’- ACCTGTCTGGTTCTGTTGGAA -3’ |
| Human *TAP2* | Forward: 5’-TAAATGCCAATGTGCTCTTG-3’  Reverse: 5’- AAGCACTTCCTGATGGCGG-3’ |
| Human *GAPDH* | Forward: 5’- TGCACCACCAACTGCTTAGC -3’  Reverse: 5’- GGCATGGACTGTGGTCATGAG -3’ |
